# Assessing the risk of diseases with epidemic and pandemic potential in a changing world

**DOI:** 10.1126/sciadv.adw6363

**Published:** 2025-07-23

**Authors:** Angela Fanelli, Alessandro Cescatti, Juan-Carlos Ciscar, Gregoire Dubois, Dolores Ibarreta, Rachel Lowe, Nicola Riccetti, Marine Robuchon, Ilaria Capua, Wojtek Szewczyk, Emanuele Massaro

**Affiliations:** ^1^ European Commission, Joint Research Centre (JRC) Scientific Development Programmes Unit, Ispra, Italy.; ^2^ European Commission, Joint Research Centre (JRC) Economics of Climate Change, Energy, and Transport Unit, Seville, Spain.; ^3^ European Commission, Joint Research Centre (JRC) Forests and Bio-Economy Unit, Ispra, Italy.; ^4^ European Commission, Joint Research Centre (JRC) Nature Conservation and Observations Unit, Ispra, Italy.; ^5^ Barcelona Supercomputing Center (BSC), Barcelona, Spain.; ^6^ Catalan Institution for Research and Advanced Studies (ICREA), Barcelona, Spain.; ^7^ Climate Change and Planetary Health and Centre for Mathematical Modelling of Infectious Diseases, London School of Hygiene and Tropical Medicine, London, UK.; ^8^ Johns Hopkins University SAIS Europe, Bologna, Italy.

## Abstract

How do human activities contribute to the emergence of zoonotic diseases that can lead to epidemics and pandemics? Our analysis of common drivers of the World Health Organization’s priority diseases suggests that climate conditions, including higher temperatures, higher annual precipitation levels, and water deficits, elevate the risk of disease outbreaks. In addition, land-use changes, human encroachment on forested areas, increased population and livestock density, and biodiversity loss contribute to this risk, with biodiversity loss showing a complex and nonlinear relationship. This study also presents a global risk map and an epidemic risk index that combines countries’ specific risk with their capacities for preparing and responding to zoonotic threats.

## Teaser

Human activities increase zoonotic disease risk.

## INTRODUCTION

Epidemics and pandemics caused by transmission of pathogens from animals to humans, zoonotic spillover, are becoming more frequent and severe, with potentially dire consequences for human health (*1*, *2*). The scientific literature points to five main groups of anthropogenic factors influencing the risk of emergence of zoonosis: climate change, human population dynamics, livestock production, agricultural intensification, and biodiversity loss. Climate change can affect the risk of infectious diseases by reshaping habitats for pathogens and their reservoirs (*3*, *4*). Livestock species often serve as hosts for zoonotic pathogens, elevating the risk of disease transmission to humans, particularly when situated near urban areas and characterized by suboptimal biosecurity and husbandry practices (*5*). Agricultural expansion and intensification have far-reaching environmental consequences, such as deforestation, defaunation, soil degradation, and water pollution (*6*), which can, in turn, affect the risk of emergence of infectious diseases. The environmental changes resulting from human activities, especially ecosystem degradation, are among the main drivers of biodiversity loss (*7*) which, in turn, affects the risk of zoonotic diseases (*8*).

Research has predominantly focused on individual diseases or specific disease groups, such as vector-borne diseases (*9*, *10*), with climate change studies primarily centering on mosquito-borne diseases (*11*). For instance, a recent study found that outbreaks of vector-borne diseases frequently occur in regions where people reside close to forests and experience climate drying (*12*). In contrast, these environmental and climate factors were not consistently associated with nonvector-borne diseases, such as Ebola and Mpox (*12*). There are also several studies that have attempted to model global risk for various emerging diseases (*1*, *13*). However, to date, a global assessment that comprehensively examines the shared drivers of zoonotic diseases with epidemic and pandemic potential has not been conducted.

The World Health Organization (WHO) maintains a list of priority diseases, namely, pathogens identified as having potential to cause severe public health emergency including epidemics and pandemics (*14*). This prioritization intends to guide global research efforts to better prepare for and mitigate potential zoonotic outbreaks. The list includes COVID-19, Crimean-Congo hemorrhagic fever (CCHF), Ebola virus disease, Lassa fever, Middle East respiratory syndrome (MERS), severe acute respiratory syndrome (SARS), Marburg virus disease (MVD), Nipah virus disease (NiV), Rift Valley fever (RVF), Zika, and a placeholder for an unknown “Disease X” (*14*, *15*). Climate change affects the risk of these diseases, including vector-borne diseases such as RVF and Zika (*16*–*19*), and nonvector-borne diseases such as Ebola, which is affected by climate change through its effect on fruit abundance, attracting bats, and increasing the chance of human contact with infected reservoirs (*20*). Extreme weather events, forest fragmentation, and deforestation can also increase the risk of disease transmission, such as Lassa fever (*21*).

Building on the above context, our study aimed to investigate the relationship between nine key human-induced drivers and the outbreak risk of WHO priority diseases, excluding COVID-19. We also developed a global risk map and an epidemic risk index that reflects each country-specific risk versus its capacity to respond to zoonotic threats.

## RESULTS AND DISCUSSION

### Locating the threat: Risk zones for the WHO priority diseases

**Fig. 1.**
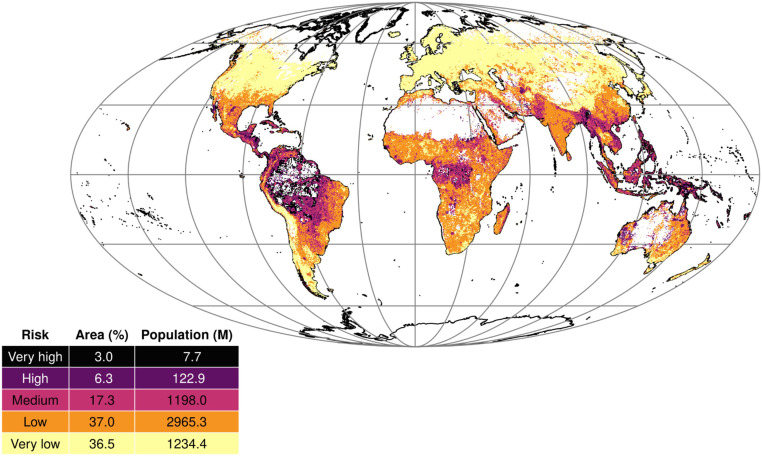
Risk of the WHO priority diseases, bias adjusted. The map displays the risk levels across different regions, with white areas indicating insufficient data for one or more predictor layers. The accompanying table provides a summary of the results, showing the percentage of land area and population [in millions (M)] falling within each risk category. The population estimate was calculated by averaging the population over the study period and summing it within each risk category.

We developed a predictive modeling framework that leveraged a spatial Bayesian additive regression trees (BART) model, a machine learning technique, to analyze outbreak data and satellite-derived variables, with the goal of identifying potential drivers and estimating the risk of WHO priority diseases. To assess the true risk, we created a proxy layer for detection probability and adjusted the observed risk predictions. Our results indicate that 9.3% of the global land surface is at high (6.3%) or very high (3%) risk, with the majority of those areas located in Latin America and Oceania (Fig. 1). The proportion of the area of each continent at high and very high risk of outbreak is the largest in Latin America (27.1%), followed by Oceania (18.6%), Asia (6.9%), Africa (5.2%), Europe (0.2%), and North America (0.08%). When accounting for the spatial distribution of the human population, we found that as much as 20% of the population lives in areas of medium risk, and 3% of the people live in areas of high and very high risk.

Our model shows good performance metrics, including high sensitivity, specificity, and overall accuracy, effectively balancing the accurate identification of both true outbreak occurrences and pseudo-absences. However, there were instances where the model predicted events that did not occur, leading to false positives. These false positives were mainly associated with areas having conditions favorable for outbreaks, even if an outbreak had not been reported or occurred there yet (refer to the Supplementary Materials for detailed metrics).

### Anthropogenic drivers influencing the risk of the priority diseases

Anthropogenic drivers influence the occurrence of certain diseases in multiple and complex ways, as a result of a combination of ecological, social, and economic pressures that combine and interact with each other to modify disease risk (*8*). We considered nine potential drivers of the WHO priority diseases, grouped into three categories: climate factors (temperature, precipitation, and water deficit), environmental factors (human-forest proximity, biodiversity loss, livestock density, and frequency of land-use change), and population (population density). In the multivariable analysis, we also incorporated the travel time to health care facilities as a bias breaker, ensuring that the probability of disease detection influenced by the proximity of health care facilities is accounted for.

#### Climate factors

**Fig. 2.**
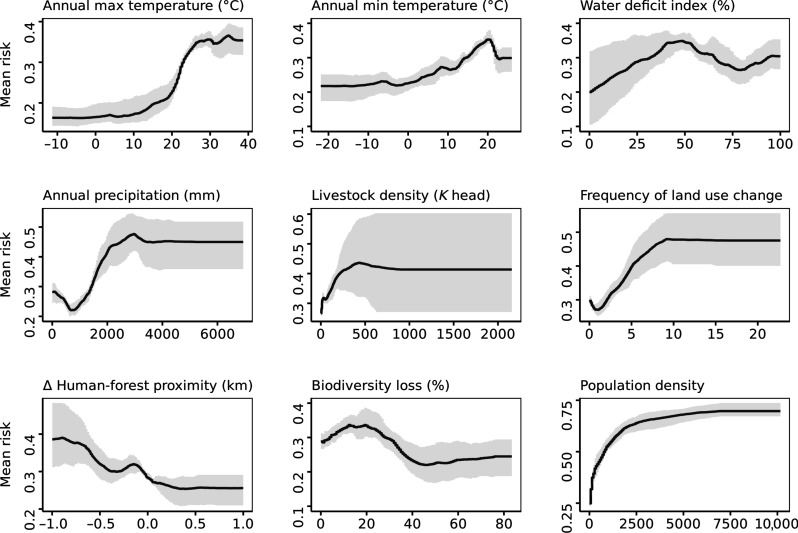
Relationship between predictor variables and the risk of WHO priority diseases. The plot illustrates the association between each predictor variable and the risk of WHO priority diseases, with uncertainty represented by the gray-shaded area. Note that all predictor variables were calculated at a spatial resolution of 30 km, using a grid-based approach.

The optimal temperature and humidity conditions for the survival of pathogens and their vectors are complex and biologically important (*22*). However, infectious diseases generally tend to thrive in higher temperatures (*23*). Our findings confirm that consistently warmer areas with higher maximum and minimum temperatures are more prone to outbreaks (Fig. 2). This observation is crucial because, as the planet warms unevenly (*24*), cooler regions may also eventually reach suitable conditions for these diseases.

Another important aspect of climate change is its impact on precipitation patterns. Our study indicates that higher annual precipitation levels can increase the risk of disease outbreaks up to a certain point, after which the risk levels off (Fig. 2). This is expected because annual precipitation is a key factor in tropical regions where these diseases are prevalent, and precipitation levels are typically high. Our findings are consistent with extensive research that links increased precipitation to a higher risk of vector-borne diseases affecting both humans and animals (*25*–*27*). In contrast, existing research on the impact of precipitation on nonvector-borne diseases remains inconclusive (*1*, *28*, *29*). Our study contributes to this body of knowledge and emphasizes the need for targeted strategies in areas experiencing changing precipitation patterns.

Annual aggregates of temperature and precipitation do not adequately capture the impact of intra-annual water availability on disease risk. The extent of water deficit influences climate and ecological conditions that affect the dynamics of host populations, vector species, and human interactions with wildlife. To address this aspect, our analysis includes the water-deficit index, representing the cumulative monthly differences between precipitation and potential evapotranspiration. This index provides valuable insights into the role of water availability throughout the year. The model results show that outbreak risk is positively correlated with water deficit levels, but not in a linear way. The highest risk occurs in areas with midlevel water-deficit index values; beyond this point, the risk decreases slightly before increasing again (Fig. 2). Water deficit can be associated with environmental stress and the resulting host density and dynamics. Scarcity of water and vegetation leads to the concentration of animals (both wildlife and livestock) around limited water and food resources (*30*), hence facilitating pathogen transmission. During periods of resource scarcity, animals may encroach upon human settlements, particularly if water deficits coincide with irrigation systems, increasing the likelihood of contact between human populations, livestock, and wildlife and subsequently elevating the risk of spillover (*31*). In addition, in water-stressed zones, the risk of contracting infectious diseases is higher because of the combined factors of poor water quality, inadequate hygiene, and insufficient sanitation resulting from water shortages (*32*). We believe that the fact that moderate to high water deficits resulted to be more conducive to outbreaks than extreme deficits may be due to the scarcity of hosts in excessively arid regions. These regions often lack sufficient vegetation and animal life, leading to fewer hosts for pathogens and lower population densities.

#### Environmental factors

High livestock density is a recognized risk factor for emerging infectious diseases, as it increases the likelihood of spillover to humans due to the increased infectious pressure from dense animal populations (*5*). Our results confirm this association, showing that higher livestock density is linked to a higher risk of outbreaks in humans. However, we found that beyond a certain density threshold, the mean risk stabilizes and becomes less predictable, highlighting the uncertainty surrounding the role of livestock density in outbreak risk (Fig. 2). This high uncertainty can be attributed to other relevant factors influencing the outbreak risk such as farming management practices, biosecurity measures, and livestock movement practices, which can either mitigate or exacerbate the risk of an outbreak, regardless of livestock density (*33*).

Expansion of pastureland for livestock is one of the primary drivers of change in land management for human needs, alongside crop intensification and urban development (*34*). Approximately half of the zoonotic emerging diseases are attributed to changes in land use, agricultural practices, food production, and wildlife hunting (*35*). Our analysis reveals that more frequent land-use changes increase the risk of an outbreak, underscoring the importance of understanding the impact of land management on disease emergence (Fig. 2).

While frequency of land-use change does not inform about specific risks associated with each type of change (e.g., agriculture to forestry), other indicators, such as human-forest proximity, can provide more insights. Our analysis shows that outbreak risk increases as the distance between humans and forests decreases (Fig. 2). Proximity to forests increases the risk of disease outbreaks by facilitating wildlife-human interactions, especially when paired with rapid population growth and urban expansion (*36*).

Land-use change affects the animal and plant species that inhabit the ecosystem, resulting in altered biodiversity. We found that the risk of an outbreak increases sharply as the ecosystem loses ~20% of its original, undisturbed species richness, but further biodiversity loss leads to a decrease in the risk, only to increase again and eventually stabilize at higher levels of biodiversity loss (Fig. 2). The role of biodiversity for the risk of emerging infectious diseases is the subject of an ongoing debate (*37*–*39*) The amplification effect hypothesis suggests that higher biodiversity can increase the abundance of certain competent host species, thereby amplifying disease transmission (*39*). The dilution effect theory, on the other hand, argues that biodiversity loss heightens disease emergence as fewer species fail to “dilute” transmission; thus, pathogens more readily infect preferred hosts, boosting disease spread (*39*). The relationship identified in this study suggests that the initial biodiversity losses lead to an increase in the risk of diseases outbreaks, de facto supporting the dilution theory. However, in our results, higher biodiversity loss was associated with a lower risk of outbreaks, which is closer to the abundance theory, or with a degree of biodiversity loss equivalent to elimination of the reservoir. A meta-analysis study (*39*) pointed out that, because of the complexity of infectious disease ecology, there is no straightforward relationship between disease risk and biodiversity. Instead, the influence of specific host species, along with their interactions with other hosts, vectors, and pathogens, appears to have a greater impact on shaping local disease risk (*39*).

#### Population

Previous studies have consistently demonstrated a positive association between human population growth and the risk of emerging infectious diseases (*1*, *13*). Our findings confirm that the impact of population density on the overall risk of disease outbreaks is substantial, outweighing the contributions of other individual factors. We observed that, when all other variables are held constant, the marginal effect of population density results in a much more pronounced risk compared to other factors (Fig. 2).

### Country epidemic risk index: Integrating risk predictions with response capacity

**Fig. 3.**
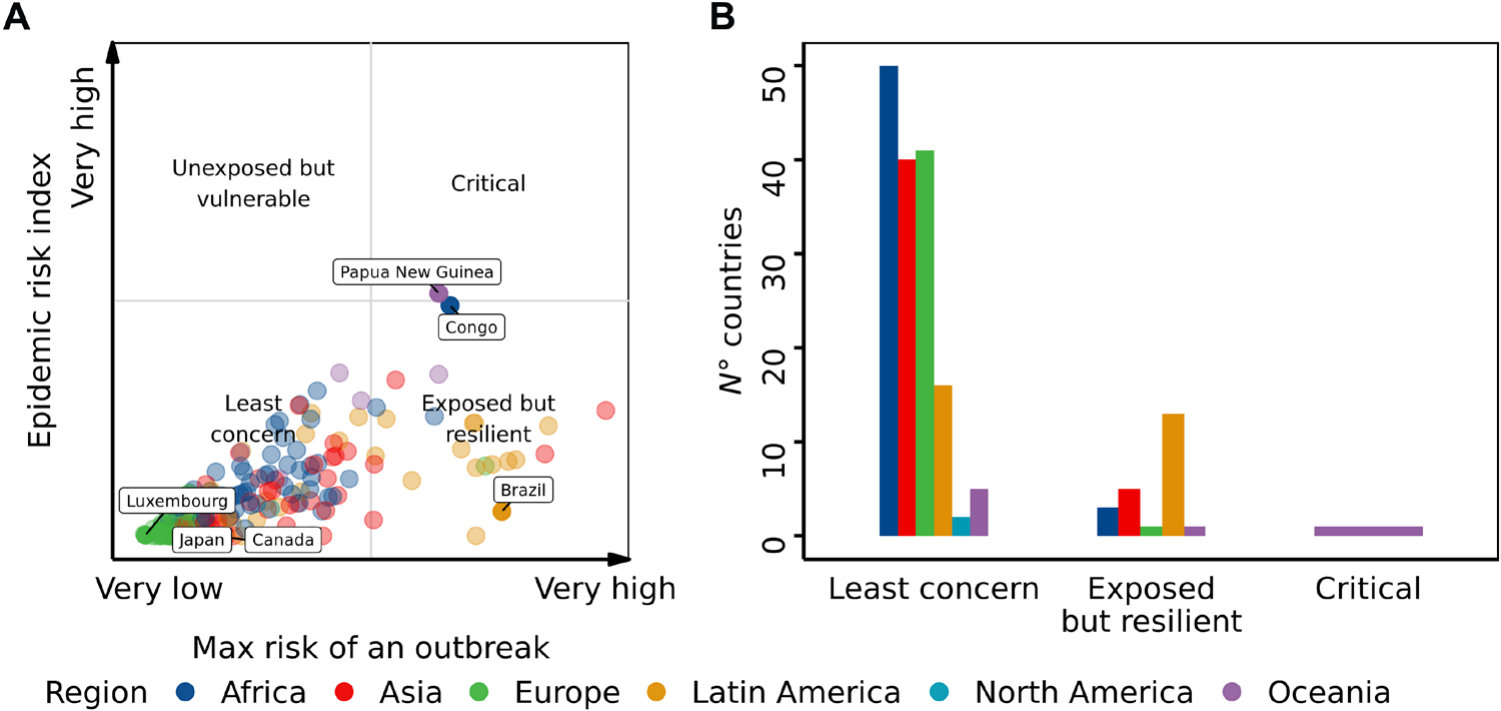
Country-level epidemic risk index, adjusted for national capacities and capabilities to respond to zoonotic events at the animal-wildlife interface. (**A**) Epidemic risk index matrix, categorized by region and is color coded, with each circle representing a country. The matrix combines maximum outbreak risk and epidemic risk, informed by C3 zoonotic event data and IHR C3 data on the human-animal health interface. (**B**) Distribution of countries across matrix categories and regions, also color coded, showing the number of countries in each category.

In this study, we developed an epidemic risk index in order to complement the risk with the capacity to respond to zoonotic outbreaks. We calculated this index by weighting the maximum risk of outbreak occurrence with country-specific data on zoonotic event response, including C3 zoonotic events and the human-animal health interface [International Health Regulations (IHR) C3] data (*40*). The index ranks Papua New Guinea and the Republic of Congo at the top (refer to the Supplementary Materials for the full ranking). Most countries are categorized as exposed but resilient, with only Papua New Guinea classified at a critical level (Fig. 3). Our findings indicate that while some countries show a linear relationship between outbreak risk and epidemic risk, others can reduce their risk through effective zoonotic response capabilities. Some countries show a marked difference between their IHR C3 data and maximum predicted outbreak risk, resulting in a lower epidemic risk index than their outbreak risk would suggest, which highlights the importance of response capacity in mitigating risk (see the Supplementary Materials). It is important to highlight that other assessments have identified the greatest vulnerabilities from West Africa to the Horn of Africa. However, these assessments used different indicators and methods (*41*).

### Closing remarks

This study highlights the critical role of environmental, climate, and sociodemographic factors in shaping the risk of diseases with epidemic and pandemic potential. By examining these interconnections, we provide insights into the drivers that increase disease risk and offer guidance for targeted prevention and intervention strategies. Our findings show that climate factors, including higher temperatures, increased precipitation, and water scarcity, are key drivers of disease outbreaks, with climate change creating new vulnerabilities as it reshapes the geographic distribution of risk. This underscores the need for continued monitoring and the integration of climate adaptation and mitigation efforts into public health planning.

Environmental changes, such as forest degradation and land-use changes, emerged as key contributors to the spread of diseases, while the role of livestock density was found to be more complex, with its impact on outbreak risk becoming less predictable beyond a certain density threshold. These findings emphasize the importance of sustainable land-use practices and conservation efforts to prevent habitat disruption that could foster conditions favorable for disease transmission. The complex relationship between biodiversity and disease risk suggests that context-specific strategies are necessary to address regional variations and account for the nonlinear nature of these interactions. Meanwhile, population density and unplanned urbanization are central to understanding disease vulnerability, particularly in densely populated areas where inadequate planning can lead to overcrowding and poor living conditions. Urban planning efforts that focus on infrastructure development, sanitation, and reducing urban inequalities can play a critical role in mitigating these risks.

Our study also demonstrates the potential of machine learning models in developing risk maps, which can help prioritize surveillance and inform public health decision-making. Translating these risk estimates into an epidemic risk index allows for the identification of high-risk areas and supports policymakers in improving response capacities, allocating resources effectively, and fostering international collaboration to address global health threats.

The methodological choices implied certain limitations that warrant further investigation. Aggregation of multiple diseases into a general model allowed identification of common risk patterns and provided a framework for policymaking; however, it led to the omission of disease-specific temporal dynamics and to the potential bias toward the diseases more prevalent in the dataset. Furthermore, we used the travel time to health care facilities as a proxy for detection bias, without accounting for the availability and quality of diagnostic services, which can affect disease reporting and diagnosis. Incorporating additional factors, such as health system capacity, quality, accessibility, and socioeconomic indicators, could further refine future models (*13*). Despite this, the current model balances data availability and predictive power, making it a useful tool for public health planning.

We also recognize that our country-level assessments align with standard practices in public health decision-making, where national-level risk assessments are used to allocate resources and guide outbreak response. While the risk of diseases can vary within countries, assessing the highest predicted risk within each country ensures that areas of concern are not overlooked. This approach remains effective for national preparedness, although future studies could benefit from integrating more granular, subnational data as they become available. Furthermore, because the IHR C3 relies on self-reported data, there is a possibility that some countries may withhold information for various reasons (*42*). Consequently, the epidemic risk index should be interpreted with caution and contextualized within the framework of available official data.

Overall, our study provides a solid foundation for understanding and mitigating the risks of epidemic and pandemic diseases. By addressing considerations related to temporal dynamics and regional variations in risk, future research can further refine predictive models and support evidence-based public health interventions. Expanding interdisciplinary collaborations and improving data collection on both human and animal outbreaks will be critical for developing adaptive, comprehensive strategies to manage emerging global health threats effectively.

## MATERIALS AND METHODS

### Risk of diseases with epidemic and pandemic potential in humans

#### Outbreaks in humans

Spatial data on outbreaks (1975–2020) were obtained from the Global Infectious Diseases and Epidemiology Network (*43*). In this dataset, outbreaks are defined as two or more linked cases of the same illness or the situation where the observed number of cases exceeds the expected number or a single case of disease caused by a pathogen that poses a significant threat to public health.. We obtained information for outbreaks of nine zoonotic diseases included in the Blueprint list of priority diseases with epidemic and pandemic potential compiled by the WHO, including CCHF, Ebola virus disease, Lassa fever, MERS, SARS, MVD, NiV, RVF, and Zika (*14*, *15*). COVID-19 was deliberately excluded because of its overwhelming prevalence compared to other diseases, which resulted in worldwide coverage and obscured the detection of patterns in the drivers. We also excluded outbreaks in hospital settings and nonautochthonous cases, as well as data lacking precise geographic reference (e.g., data reported at country level). Georeferenced data (*n* = 131) were gridded into a 30-km raster based on the Mollweide projection. The final dataset comprises 115 locations (i.e., 30-km–by–30-km cells) where at least one outbreak occurred. The relatively large cell size was motivated by both the resolution of the rasters and human mobility considerations. We considered that the spatial point of an outbreak does not necessarily coincide with the point of infection, as the latter may have occurred within a radius that defines surrounding areas around the locations.

#### Predictor variables

We compiled a dataset of nine predictor variables reflecting hypothesized drivers of disease emergence. All predictors were transformed into a grid of 30-km spatial resolution. These covariates are categorized into three main groups: (i) climate (annual maximum temperature, annual minimum temperature, annual precipitation, and water-deficit index), (ii) environment (livestock density, change frequency of land use, human-forest proximity, and biodiversity loss), and (iii) population (population density) (Table 1). Annual maximum and minimum temperatures were calculated by first averaging the monthly data to obtain yearly averages and then averaging these yearly averages over the study period to determine the overall average conditions. The water-deficit index calculation follows the approach of Title and Bemmels (*44*), and it is based on monthly temperature, precipitation, and solar radiation data from 1975 to 2020 (*45*). For precipitation, we calculated total annual estimates by summing the monthly values and then computed the average of these totals over the duration of the study period. Using long-term average climate conditions, we aimed to capture the average conditions that are favorable for disease emergence, acknowledging that areas that do not yet meet these conditions may eventually reach them because of ongoing climate change. This approach enables us to identify regions that are currently suitable for disease transmission, as well as the conditions that will make other areas suitable in the future as the climate continues changing. Livestock density combines densities of buffaloes, cattle, chickens, ducks, goats, horses, pigs, and sheep (head per square kilometer or birds per square kilometer) into a single predictor. The information was retrieved from the Gridded Livestock of the World dataset (*46*). The change in frequency of land use between 1960 and 2019 is based on the global Historic Land Dynamics Assessment and considers six generic land-use categories: urban areas, cropland, pasture/rangeland, forest, unmanaged grass/shrub land, and sparse/no vegetation (*47*). The human-forest proximity, a proxy for the change in human-wildlife interactions, was calculated as a share-weighted sum of the distance between each pixel’s population and the nearest forest. The share of the population in each pixel within each 30-km cell, compared to the total population of the cell, was used as weights. Last, the difference in the proximity index over the study period was computed as in Eq. 1

$$∆{\text{HFC}}_{i}\propto \frac{\sum_{i}^{t_{1}}d_{i}\times {\text{POP}}_{i}}{\sum_{i}^{t_{1}}{\text{POP}}_{i}}-\frac{\sum_{i}^{t_{0}}d_{i}\times {\text{POP}}_{i}}{\sum_{i}^{t_{0}}{\text{POP}}_{i}}$$ (1)

**Table 1.** Description of explanatory predictor variables. Set of explanatory predictors is included.

Category (theme)	Variable name	Description of variable (including calculation and definition)	Temporal coverage (start year–end year)	Source spatial resolution (°)^*^	Source reference system	Source
Climate	Annual maximum temperature	Average of the annual maximum temperature (°C)	1975–2020	0.0416°	WGS84 EPSG:4326	(*45*)
Climate	Annual minimum temperature	Average of the annual minimum temperature (°C)	1975–2020	0.0416°	WGS84 EPSG:4326	(*45*)
Climate	Water-deficit index	An index comparing precipitation to water demands for evaporation and transpiration (%)	1975–2020	0.0416°	WGS84 EPSG:4326	(*45*)
Climate	Annual total precipitation	Average of the annual total precipitation (mm)	1975–2020	0.0416°	WGS84 EPSG:4326	(*45*)
Environment	Livestock density	Number of livestock heads	2015	0.083°	WGS84 EPSG:4326	(*46*)
Environment	Frequency of land-use change	Number of times the land-use category changed	1960–2019	0.083°	WGS84 EPSG:4326	(*47*)
Environment	ΔHuman-forest proximity	The change in human-forest proximity between 1975 and 2020 (km)	1975–2020	0.083°	WGS84 EPSG:4326	(*47*, *49*)
Environment	Biodiversity loss	The percentage of pristine species richness lost in response to land-use intensity (%)	2010	0.083°	WGS84 EPSG:4326	(*48*)
Population	Population density	Average of the number of inhabitants per cell throughout the study period	1975–2020	0.083°	WGS84 EPSG:4326	(*49*)
Detection (bias breaker)	Travel time to health care facilities	Average time needed to reach the closest health care facility via both walking and motorized transport (min)	2019	0.083°	WGS84 EPSG:4326	(*50*)

*0.0416° ≈ 4.6 km (at the equator) and 0.083° ≈ 9.2 km (at the equator).

where POP denotes the population density, *d* the distance to the nearest forest, $t_{0}$ is 1975, $t_{1}$ is 2020, and i is the cell. Lower values of this change variable characterize areas where the population density increased and the distance to forest decreased between 1975 and 2020, thus denoting potentially intensifying contact between humans and wildlife. The biodiversity loss is introduced as a time-invariant variable based on the mean output of two models with different land-use intensity indicators, and it is expressed as percentage of the pristine species richness lost (*48*). We initially anticipated that the geographic distribution of outbreaks would closely follow the distribution of the human population. To account for this, we included population density as a covariate in our model. Population density was calculated by averaging the number of inhabitants per spatial unit (cell) over the entire study period, providing a comprehensive representation of human settlement patterns (*49*). Our hypothesis is that the data may be subject to detection biases, which arise from variations in proximity to and accessibility of health care services, potentially influencing the observed outbreak distribution. To account for detection probability, we included the travel time to health care facilities in our model framework (see the “Detection bias” section). Travel time to health care facilities was estimated by averaging the walking and motorized travel time rasters developed by Weiss *et al.* (*50*).

#### Modeling framework

The adopted model framework is a BART model (*51*), a Bayesian approach to classification and regression trees. In this study, the BART model is implemented using locations where at least one human outbreak was reported. To account for the absence of outbreaks, pseudo-absences (artificial absence locations) were generated at sites where no outbreaks occurred. We created 10 random subsets of pseudo-absence locations, each containing twice the number of records as the corresponding outbreak datasets (i.e., each of them containing 115 presence and 230 pseudo-absences). This approach allowed us to assess the potential impact of varying pseudo-absence sets on the dependent variable (*52*). Therefore, the model can be considered an ensemble classification model resulting from the combination (average) of 10 models built with different sets of pseudo-absence locations.

We constructed three ensemble models, each comprising 10 individual models:

1) The first model (“model framework 1”) included all nine predictors to estimate the marginal effect, while accounting for potential biases in detection.

2) The second model (“model framework 2”) considered the nine risk factors, excluding the travel time to health care facilities to predict the observed risk.

3) The third model (“model framework 3”) used the travel time to health care facilities as the sole variable to produce a layer that could be used to correct the observed risk and estimate the true risk.

The steps implemented for each individual BART model within the set of 10 models of a framework were as follows:

1) Build the BART model with 200 trees, 1000 posterior draws, and 10 burn-in iterations.

2) Calculate for each cell the mean spatial prediction as the average of the successive sum-of-trees model draws.

3) Summarize posterior uncertainty by calculating the cell-wise SD.

4) Validate the model with a 3-fold cross-validation approach and compute the area under the curve, which measures overall discrimination capacity and threshold-dependent metrics, considering the threshold that maximizes the distance to the identity line [e.g., maximum(Se + Sp)] (*53*).

All estimates were calculated as the average across all 10 models, while the validation metrics were firstly averaged across the three folds for each individual model and lastly averaged across all 10 models in the ensemble to derive an overall estimate.

#### Relationship between anthropogenic drivers and outbreak risk

For the first framework, we calculated the marginal effect of each predictor on the predicted outcome (i.e., probability of an outbreak) by applying a partial dependence function (*54*). By averaging out the effects of all other features, we derived a function that isolates the relationship between the target variable and the outcome. We then created partial dependence plots to illustrate the marginal effect of specific features on model predictions, allowing us to examine how changes in these features affect the outcome. This approach provides a simplified representation of the complex interactions within the model, making it easier to interpret the relationship between the feature of interest and the predicted outcome. Marginal effects for each predictor were estimated for the full range of each predictor. Effects were calculated for individual variables and subsequently averaged to derive a mean risk value across the model ensemble. In addition, for each model, marginal effects were quantified at specific quantiles for the posterior prediction distribution (0.025, 0.1, 0.2, 0.3, 0.4, 0.5, 0.6, 0.7, 0.8, 0.9, and 0.975) to construct 95% confidence intervals.

#### Detection bias

The geographic distribution of outbreaks is likely influenced by inconsistent detection and reporting. A common approach in epidemiology to assess the relationship between risk factors and outcome while addressing confounding and selection bias is to construct a multivariable model. This method controls for confounding and bias by investigating the factors of interest while keeping potential confounders constant (*55*). On the basis of the above, as part of our analysis, we included the travel time to health care facilities as a covariate in one of our model frameworks. We subsequently applied a partial dependence function, as outlined in the “Relationship between anthropogenic drivers and outbreak risk” section, to assess the effect of each individual covariate on the predicted outcome. By averaging out the effects of all other features, we derived a function that isolates the relationship between the target variable and the outcome. To build bias-corrected risk maps, researchers have developed various proxies to estimate reporting effort. For instance, some studies have used the locations of sampling sites to estimate “sampling effort” (*56*). Others have analyzed the affiliations of authors in the Journal of Infectious Diseases to gauge “reporting effort” (*1*). Furthermore, PubMed searches for specific keywords by country have been used to assess “reporting bias” and quantify the relative volume of published research (*57*). As an alternative to traditional proxy-based approaches, Allen *et al.* (*13*) developed a modeling framework that used a boosted regression tree model to estimate reporting effort. This model incorporated a suite of predictors, including human population, accessibility, urbanized land, disability-adjusted life year rates, health expenditure, and gross domestic product. The output of this model was used to create a layer representing reporting effort (*13*). We adopted a similar approach by developing a model (see the “Modeling framework” section for details) that uses travel time to health care facilities as the sole predictor to estimate the likelihood of outbreak occurrence. The output of this model represents the probability of an outbreak being detected given its proximity to a health care facility, effectively capturing the spatially heterogeneous detection probability. This was used to calculate the true risk predictions (see the “True risk predictions” section).

#### True risk predictions

We assumed that the distribution of observed outbreaks was conditional on the detection probability, which is influenced by health care accessibility. Using our second model framework, we estimated the observed risk relative to the observed distribution of outbreaks, considering the nine hypothesized risk factors while excluding travel time to health care facilities. By quantifying the detection bias through the probability of detection (output of third model framework), we were able to estimate the true underlying distribution of outbreaks by adjusting the observed risk for the bias based on Eq. 2. The true risk layer was then rescaled between 0 and 1

$$\text{True risk}\propto \frac{\text{Observed risk}}{\text{Detection bias}}$$ (2)

This approach is analogous to factoring out survey effort in ecological studies, where observed data are weighted by the measured survey effort, assuming a uniform ideal distribution of search effort across the landscape (*58*). Our approach is similar to that of Allen *et al.* (*13*), who used a more complex layer representing reporting effort to adjust predictions. However, we opted for a simpler approach, focusing solely on detection bias, as we found that this variable had a profound impact on the outbreak distribution.

Once the true risk was estimated, we computed the proportion of cells at different levels of risk for both the entire globe and each region. We reclassified the cell values into five classes: very low (*x* < 0.067), low (0.067 ≤ *x* < 0.138), medium (0.138 ≤ *x* < 0.236), high (0.236 ≤ *x* < 0.401), and very high (0.401 ≤ *x* ≤ 1). The Fisher’s algorithm was used to create groups with maximized homogeneity (*59*), ensuring that the classes were as distinct and consistent as possible. For comparison, we then applied the same categorization to the observed risk, allowing us to assess the differences between the true and observed risk distributions.

#### Country epidemic risk index

To calculate the epidemic risk index, we adjusted the true risk predictions by the capacities and capabilities of countries to respond to zoonotic events at the animal-wildlife interface. Data on the IHR C3 indicator were obtained for the year 2018, 2019, and 2020 from the States Parties Self-Assessment Annual Reporting Tool, which is based on the IHR (*40*). The IHR C3 is available at country level, and it is an indicator reflecting collaborative efforts in addressing zoonoses, encompassing a country’s ability to prepare for, prevent, identify, conduct risk assessments for, and report health concerns at the human-animal interface. The maximum value of the risk within each country was computed and then adjusted by the average IHR C3 (Eq. 3). This approach captures the vulnerability to zoonotic events originating from animals. Consequently, it enables the identification of the most vulnerable areas, which are those characterized by higher values

$${\text{ER}}_{c}\propto \text{max}\left(y_{i}\right)\times \left(1-\hat{\text{IHR}\;C3_{c}}\right)$$ (3)

where $\hat{y}$ denotes the average fitted value for cell i (i.e., average risk prediction across the 100 BART models), and $\hat{\text{IHR}\;C3_{c}}$ is the average IHR C3 indicator for country *c* from 2018 to 2020.

Last, we created a matrix by integrating the maximum risk of an outbreak and the epidemic risk index to categorize countries into four distinct categories: least concern (both the maximum risk of an outbreak and the epidemic risk index are ≤0.5), unexposed but vulnerable (the maximum risk of an outbreak is ≤0.5, and the epidemic risk index is >0.5), exposed but resilient (the maximum risk of an outbreak is >0.5, but the epidemic risk index is ≤0.5), and critical (the maximum risk of an outbreak and the epidemic risk index are >0.5).

### Software

All the analyses were performed using R software version 4.3.2 (*60*). The BART models were fitted using the R package dbarts (*61*).

## Supplementary Materials

### This PDF file includes:

Figs. S1 to S13 Tables S1 and S2 Supplementary Text References
