# Supplementary material for: Assessing the risk of diseases with epidemic and pandemic potential in a changing world
Source: Sci Adv. 2025 Jul 23;11(30):eadw6363. doi: 10.1126/sciadv.adw6363 (PMC12285694; doi:10.1126/sciadv.adw6363)
Supplement: Supplementary file 1 — Figs. S1 to S13 Tables S1 and S2 Supplementary Text References [file sciadv.adw6363_sm.pdf]

Science Advances
AAAS

Supplementary Materials for

Assessing the risk of diseases with epidemic and pandemic potential in a changing world

Angela Fanelli *et al.*

Corresponding author: Angela Fanelli, angela.fanelli@ec.europa.eu

Sci. Adv. **11**, eadw6363 (2025)
DOI: 10.1126/sciadv.adw6363

This PDF file includes:

Figs. S1 to S13
Tables S1 and S2
Supplementary Text
References

Additional figures and tables

We identified 131 georeferenced records of outbreaks of infectious diseases with epidemic and pandemic potential that occurred between 1975 and 2020. By converting these points into a 30-by-30 km grid raster format, we grouped multiple outbreaks within each cell as a single location. This process reduced the number of unique outbreak locations from 131 to 115.

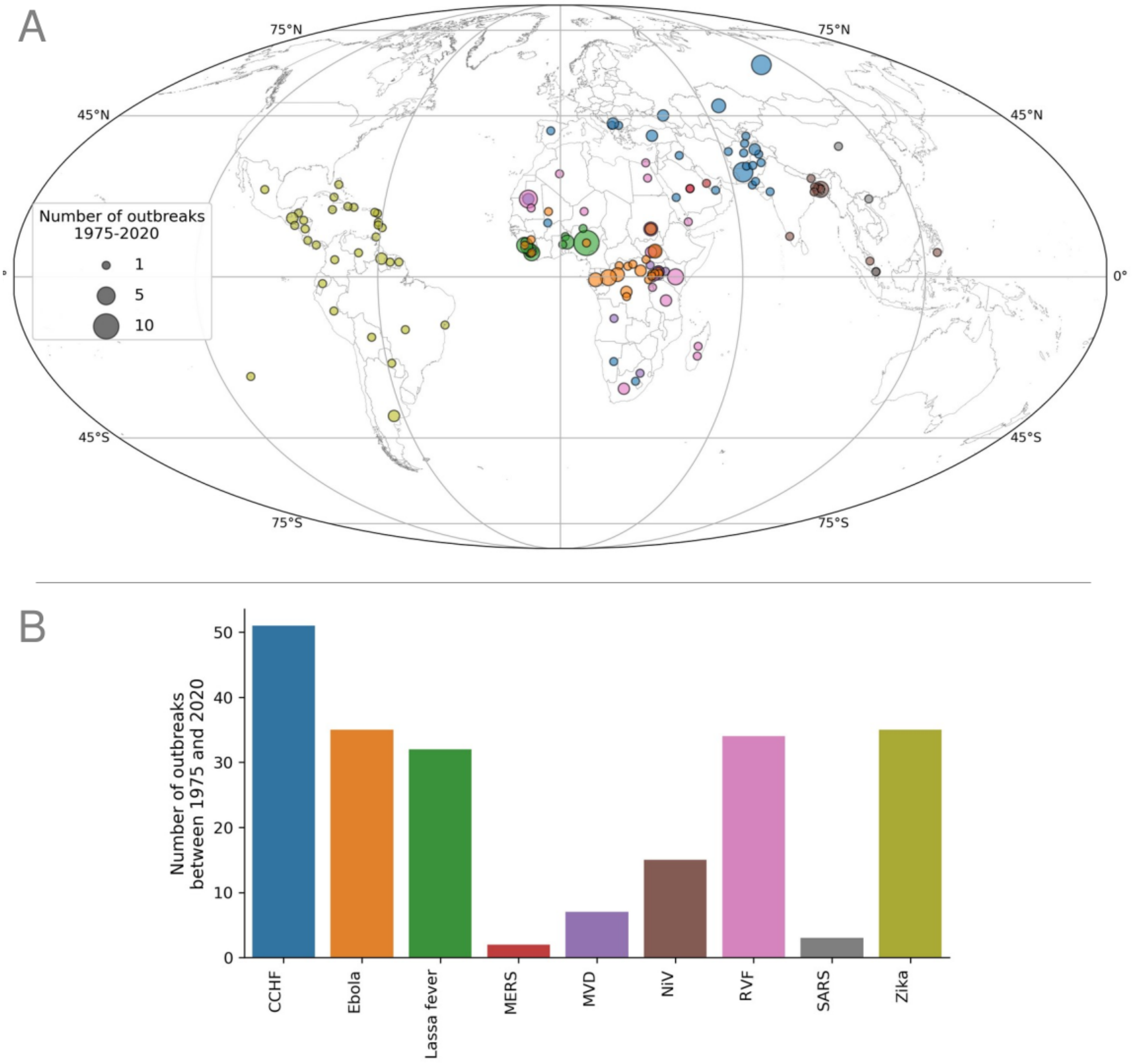

Fig. S1: Global distribution of outbreaks caused by diseases with epidemic and pandemic potential: Crimean-Congo haemorrhagic fever (CCHF), Ebola virus disease, Lassa fever, Middle East respiratory syndrome (MERS), Severe acute respiratory syndrome (SARS), Marburg virus disease (MVD), Nipah virus (NiV) disease, Rift Valley Fever (RVF), and Zika, as identified by the World Health Organization (WHO) in their most recent review of priority diseases.

(A) *Geographic distribution of disease outbreaks with epidemic and pandemic potential in humans between 1975 and 2020, mapped by longitude and latitude.* ***(B)*** *Frequency of outbreak locations, showing the number of unique spatial points (defined by longitude and latitude) where these diseases were reported in humans during the same period.*

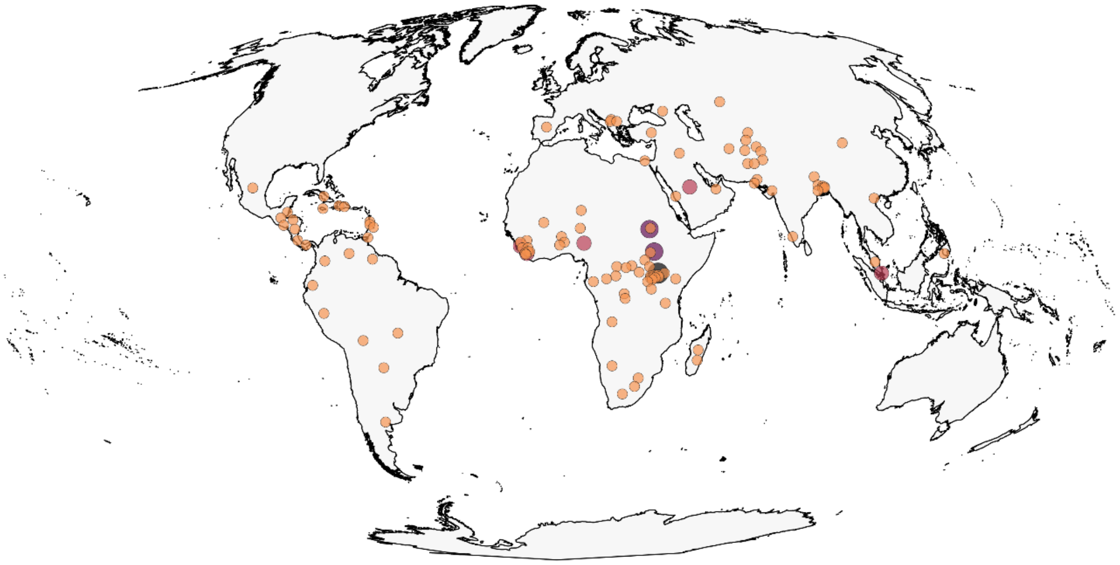

Fig. S2: Disease richness map.

Number of different diseases causing outbreaks within each cell: dot size and colour represent the number of different diseases causing outbreaks within each cell, with a range of 1 to 4 diseases. The colour scale transitions from orange (1 disease) to black (4 diseases), and dot size is proportionate to the disease richness, providing a visual representation of the diversity of outbreak-causing diseases in each area

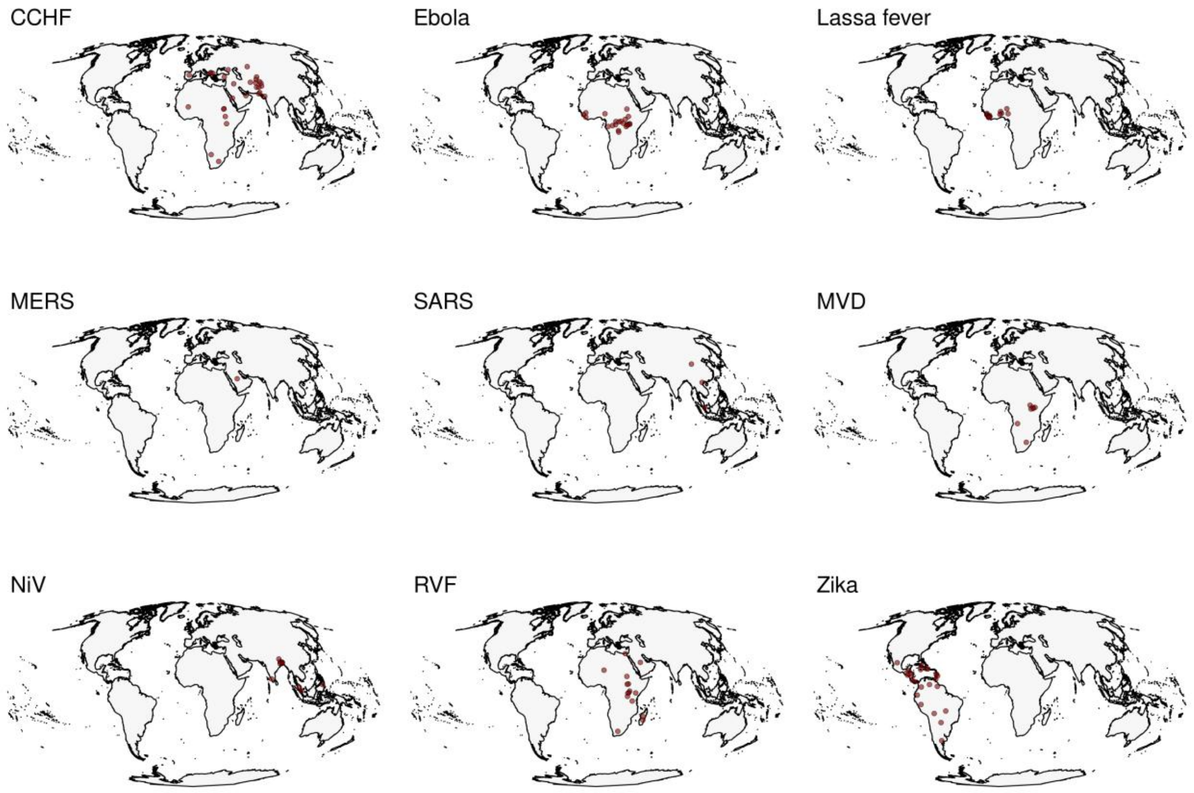

Fig. S3: Disease presence distribution.

Distribution of disease presence at a unique location in a 30-by-30 km cell grid.

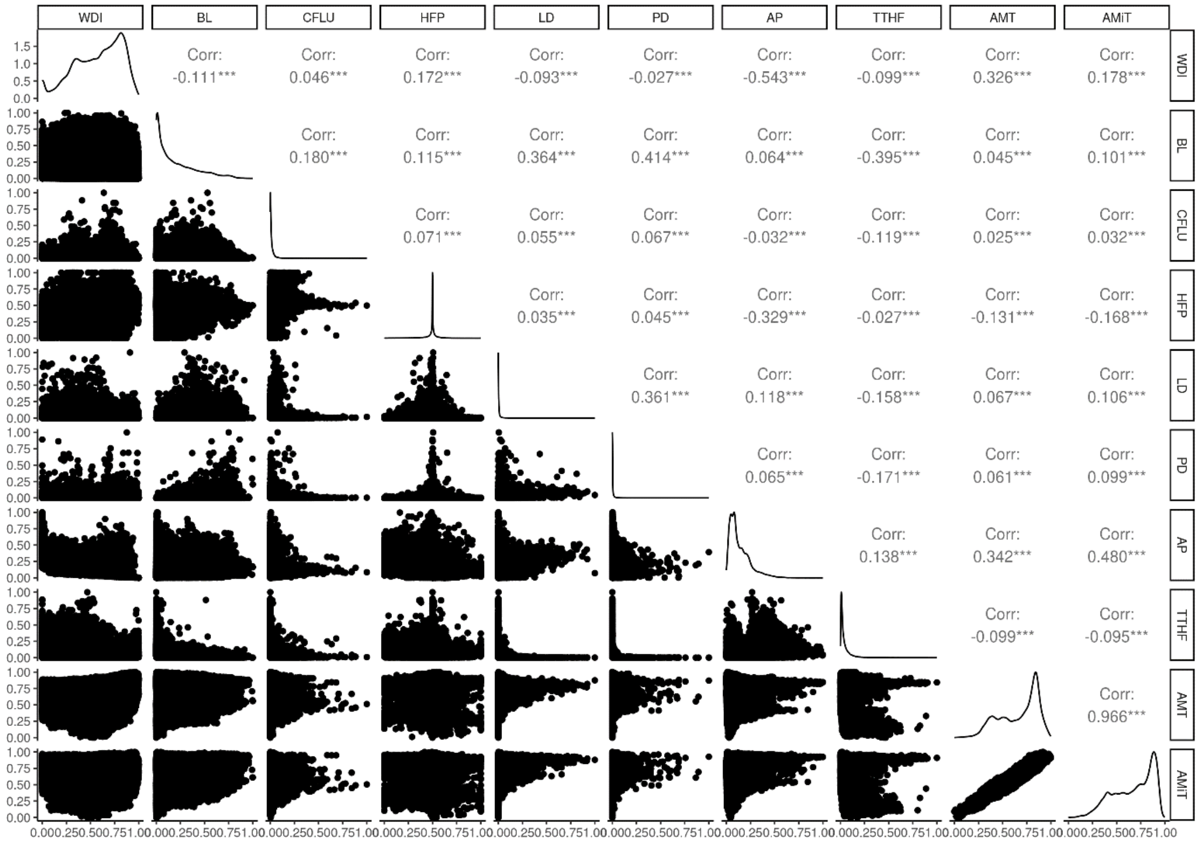

Fig. S4: Predictor variable correlations Scatterplot matrix and correlation coefficients for predictors in the models.

For visualization purpose, values were rescaled between 0 and 1. Variables Represented in the Figure: WDI (water deficit index), BL (biodiversity loss), CFLU (change frequency in land use), HFP (human-forest proximity), LD (livestock density), PD (population density), AP (annual precipitation), TTHF (travel time to healthcare facilities), AMT (annual maximum temperature), AMIT (annual minimum temperature).

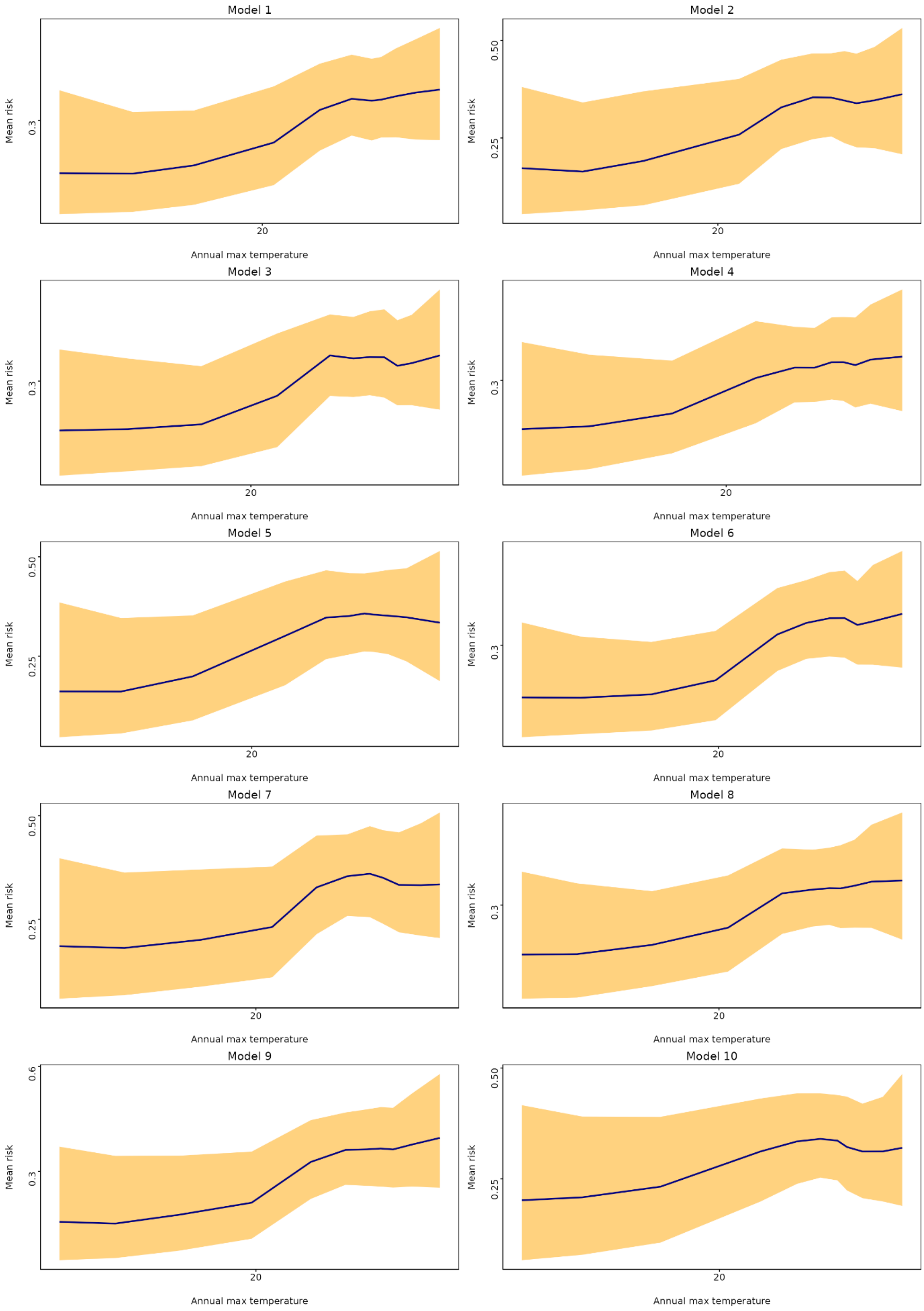

Model 1
Mean risk
0.3
20
Annual max temperature
Model 2
Mean risk
0.50
0.25
20
Annual max temperature
Model 3
Mean risk
0.3
20
Annual max temperature
Model 4
Mean risk
0.3
20
Annual max temperature
Model 5
Mean risk
0.50
0.25
20
Annual max temperature
Model 6
Mean risk
0.3
20
Annual max temperature
Model 7
Mean risk
0.50
0.25
20
Annual max temperature
Model 8
Mean risk
0.3
20
Annual max temperature
Model 9
Mean risk
0.6
0.3
20
Annual max temperature
Model 10
Mean risk
0.50
0.25
20
Annual max temperature

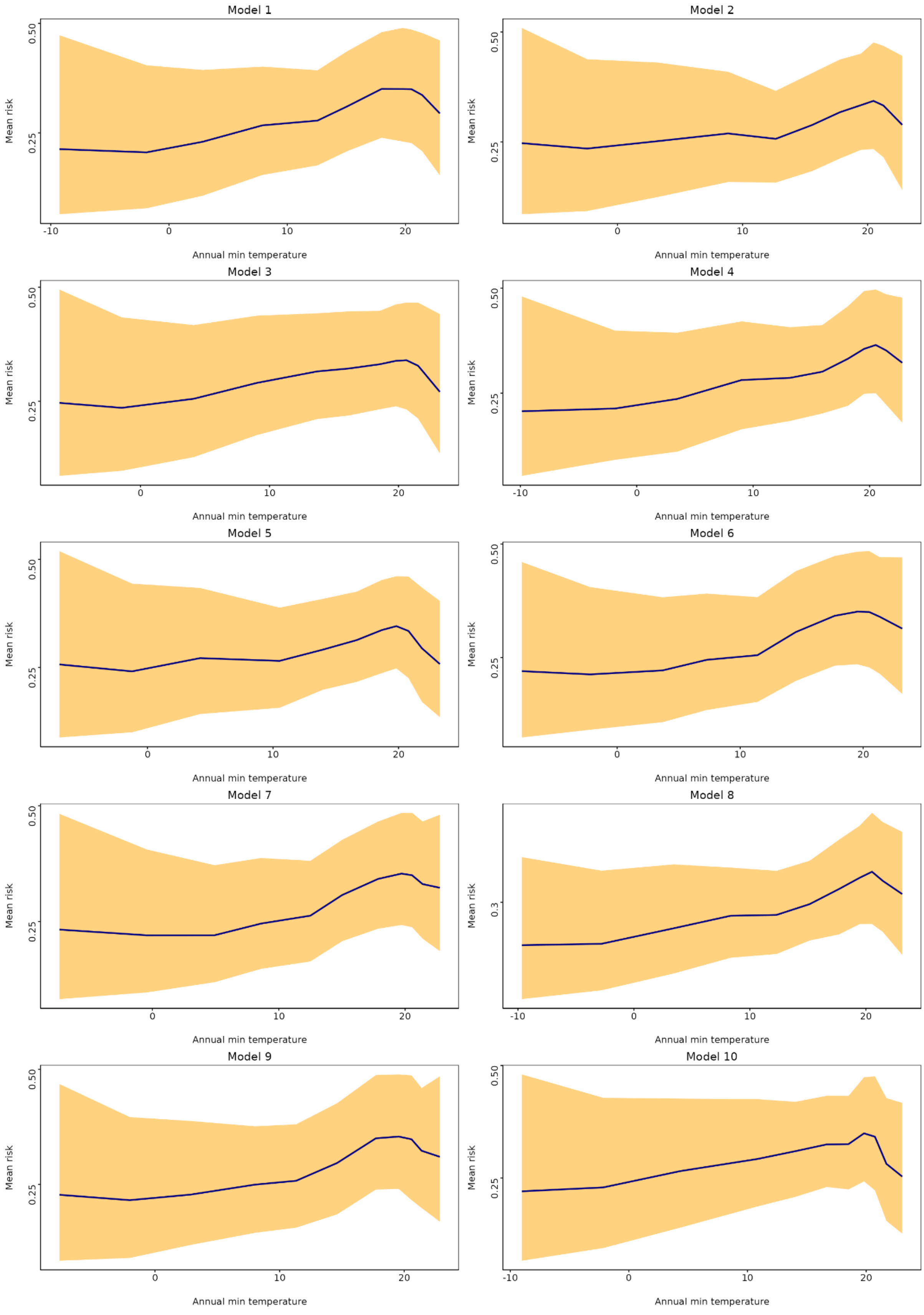
Model 1
Mean risk
0.50
0.25
-10
0
10
20
Annual min temperature
Model 2
Mean risk
0.50
0.25
0
10
20
Annual min temperature
Model 3
Mean risk
0.50
0.25
0
10
20
Annual min temperature
Model 4
Mean risk
0.50
0.25
-10
0
10
20
Annual min temperature
Model 5
Mean risk
0.50
0.25
0
10
20
Annual min temperature
Model 6
Mean risk
0.50
0.25
0
10
20
Annual min temperature
Model 7
Mean risk
0.50
0.25
0
10
20
Annual min temperature
Model 8
Mean risk
0.3
-10
0
10
20
Annual min temperature
Model 9
Mean risk
0.50
0.25
0
10
20
Annual min temperature
Model 10
Mean risk
0.50
0.25
-10
0
10
20
Annual min temperature

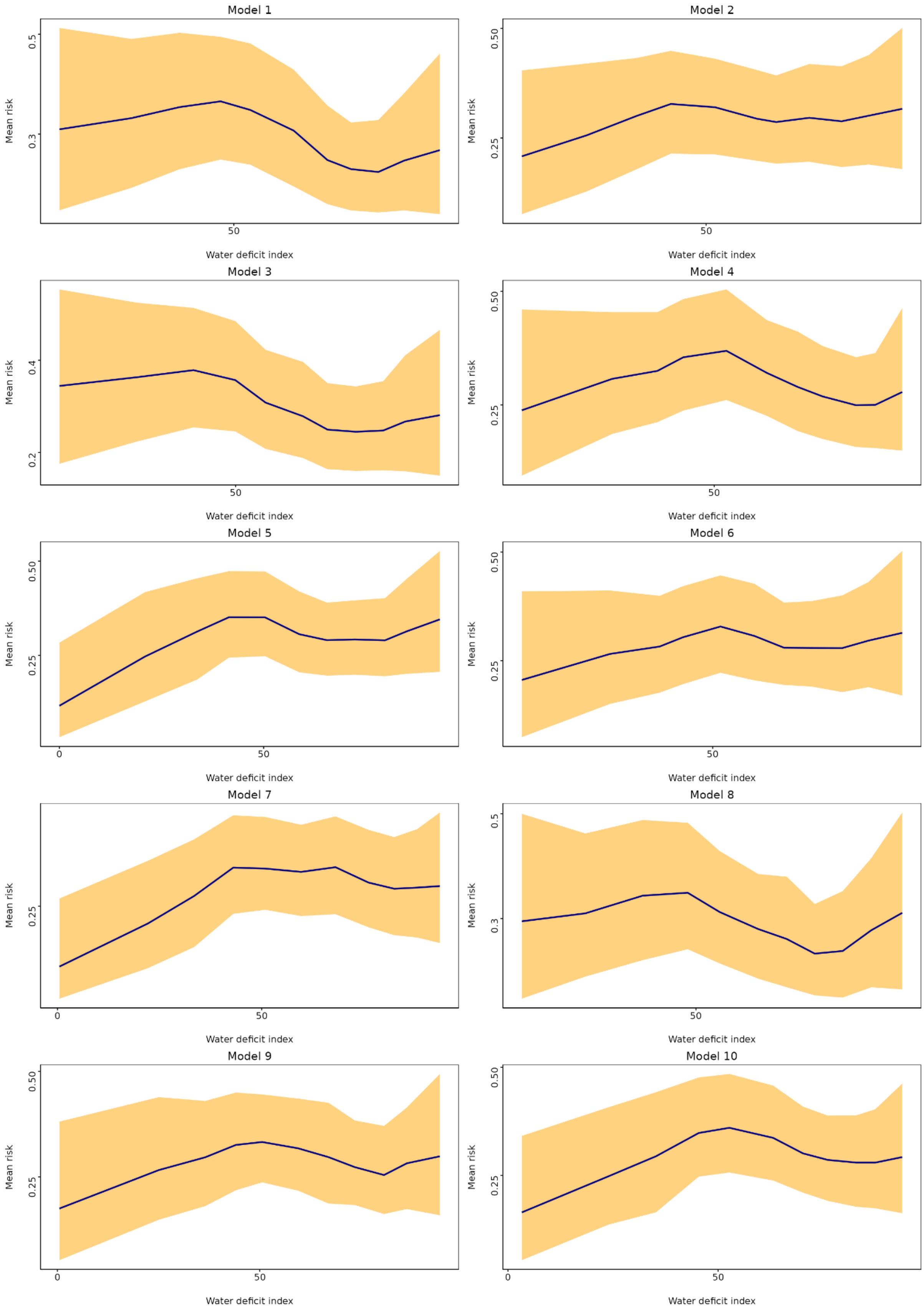
Model 1
Mean risk
0.5
0.3
50
Water deficit index
Model 2
Mean risk
0.50
0.25
50
Water deficit index
Model 3
Mean risk
0.4
0.2
50
Water deficit index
Model 4
Mean risk
0.50
0.25
50
Water deficit index
Model 5
Mean risk
0.50
0.25
0
50
Water deficit index
Model 6
Mean risk
0.50
0.25
50
Water deficit index
Model 7
Mean risk
0.25
0
50
Water deficit index
Model 8
Mean risk
0.5
0.3
50
Water deficit index
Model 9
Mean risk
0.50
0.25
0
50
Water deficit index
Model 10
Mean risk
0.50
0.25
0
50
Water deficit index

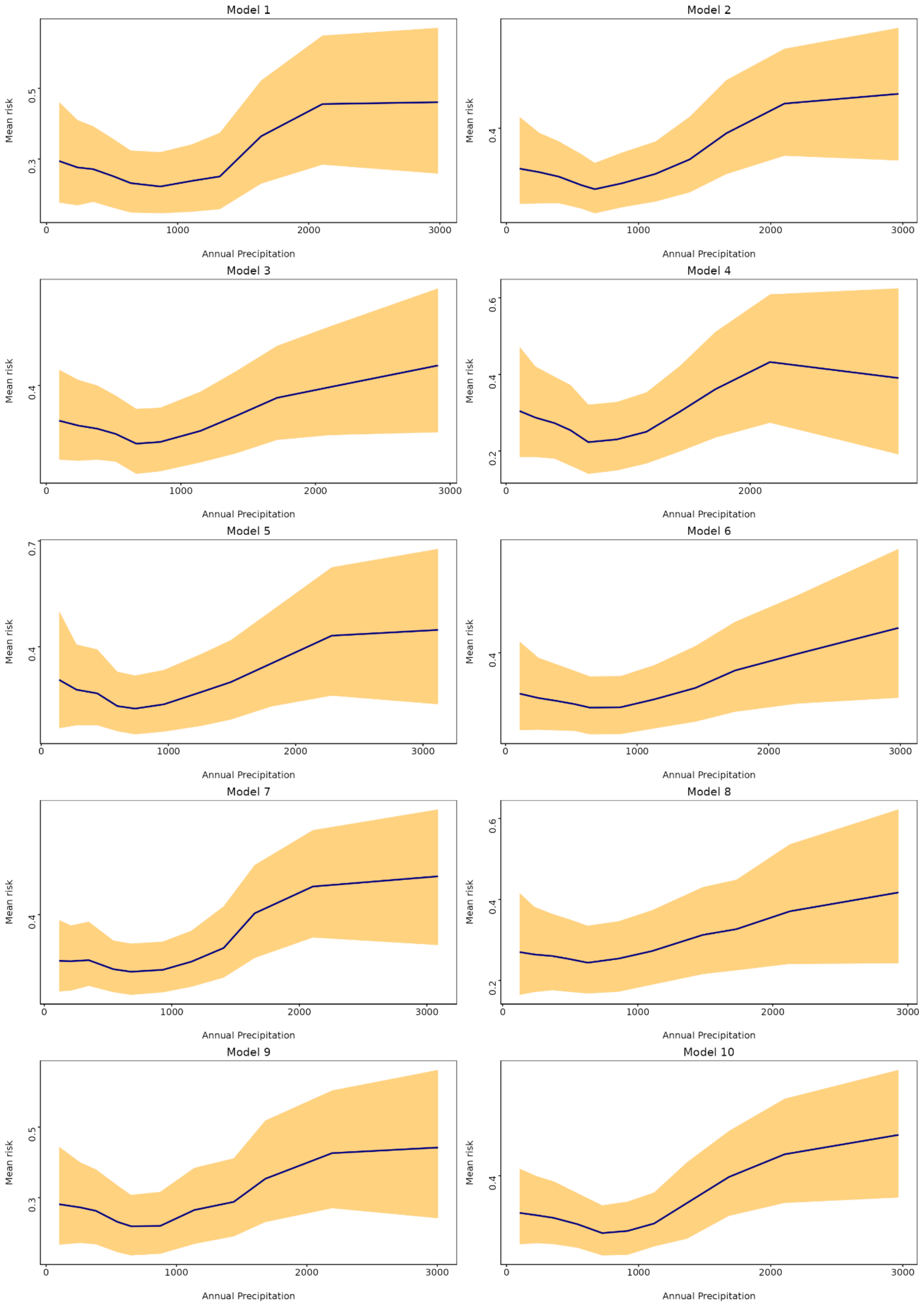
Model 1
Mean risk
0.5
0.3
0
1000
2000
3000
Annual Precipitation
Model 2
Mean risk
0.4
0
1000
2000
3000
Annual Precipitation
Model 3
Mean risk
0.4
0
1000
2000
3000
Annual Precipitation
Model 4
Mean risk
0.6
0.4
0.2
0
2000
Annual Precipitation
Model 5
Mean risk
0.7
0.4
0
1000
2000
3000
Annual Precipitation
Model 6
Mean risk
0.4
0
1000
2000
3000
Annual Precipitation
Model 7
Mean risk
0.4
0
1000
2000
3000
Annual Precipitation
Model 8
Mean risk
0.6
0.4
0.2
0
1000
2000
3000
Annual Precipitation
Model 9
Mean risk
0.5
0.3
0
1000
2000
3000
Annual Precipitation
Model 10
Mean risk
0.4
0
1000
2000
3000
Annual Precipitation

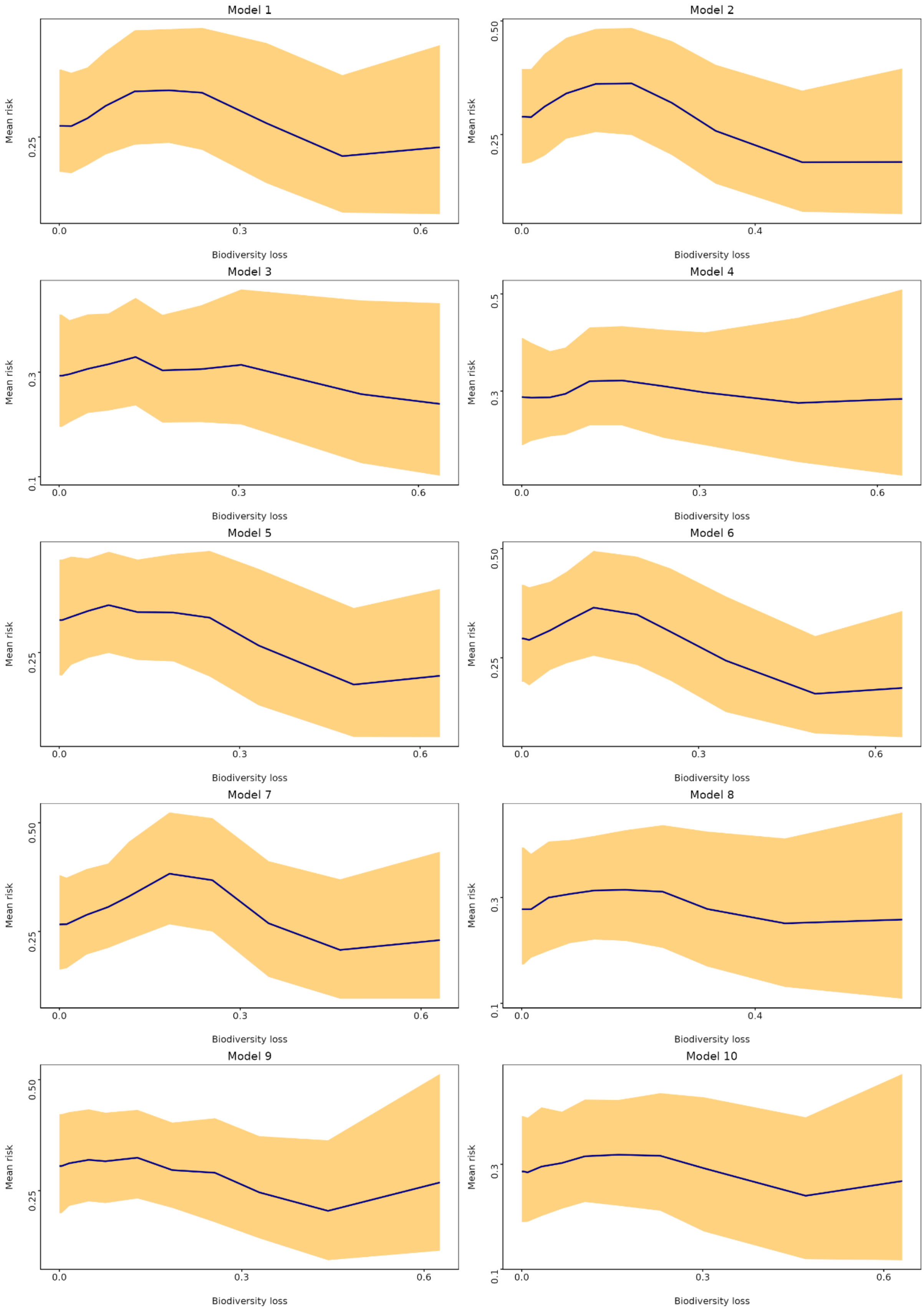

Model 1
Mean risk
0.25
0.0
0.3
0.6
Biodiversity loss
Model 2
Mean risk
0.50
0.25
0.0
0.4
Biodiversity loss
Model 3
Mean risk
0.3
0.1
0.0
0.3
0.6
Biodiversity loss
Model 4
Mean risk
0.5
0.3
0.0
0.3
0.6
Biodiversity loss
Model 5
Mean risk
0.25
0.0
0.3
0.6
Biodiversity loss
Model 6
Mean risk
0.50
0.25
0.0
0.3
0.6
Biodiversity loss
Model 7
Mean risk
0.50
0.25
0.0
0.3
0.6
Biodiversity loss
Model 8
Mean risk
0.3
0.1
0.0
0.4
Biodiversity loss
Model 9
Mean risk
0.50
0.25
0.0
0.3
0.6
Biodiversity loss
Model 10
Mean risk
0.3
0.1
0.0
0.3
0.6
Biodiversity loss

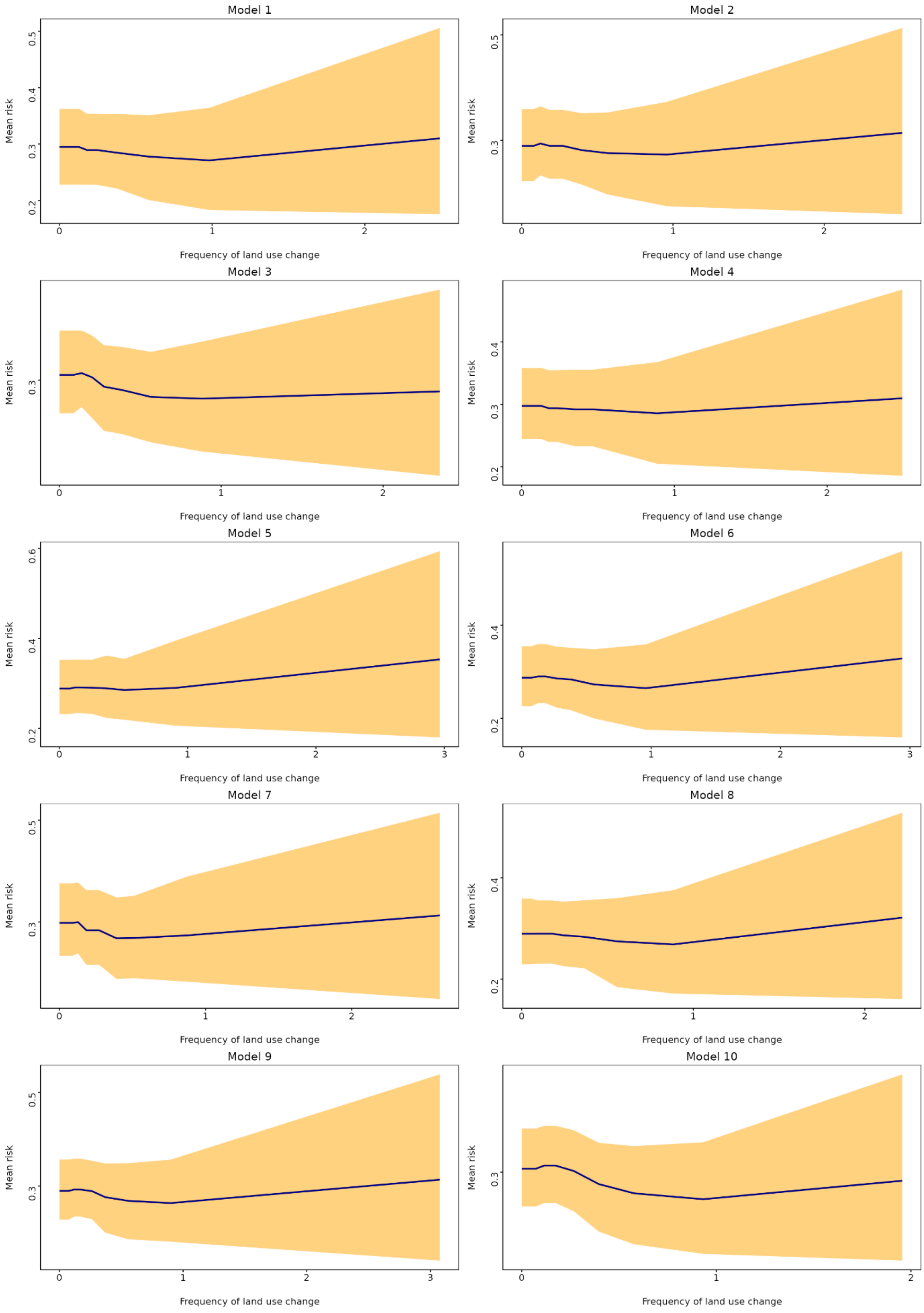
Model 1
Mean risk
0.2
0.3
0.4
0.5
0
1
2
Frequency of land use change
Model 2
Mean risk
0.3
0.5
0
1
2
Frequency of land use change
Model 3
Mean risk
0.3
0
1
2
Frequency of land use change
Model 4
Mean risk
0.2
0.3
0.4
0
1
2
Frequency of land use change
Model 5
Mean risk
0.2
0.4
0.6
0
1
2
3
Frequency of land use change
Model 6
Mean risk
0.2
0.4
0
1
2
3
Frequency of land use change
Model 7
Mean risk
0.3
0.5
0
1
2
Frequency of land use change
Model 8
Mean risk
0.2
0.4
0
1
2
Frequency of land use change
Model 9
Mean risk
0.3
0.5
0
1
2
3
Frequency of land use change
Model 10
Mean risk
0.3
0
1
2
Frequency of land use change

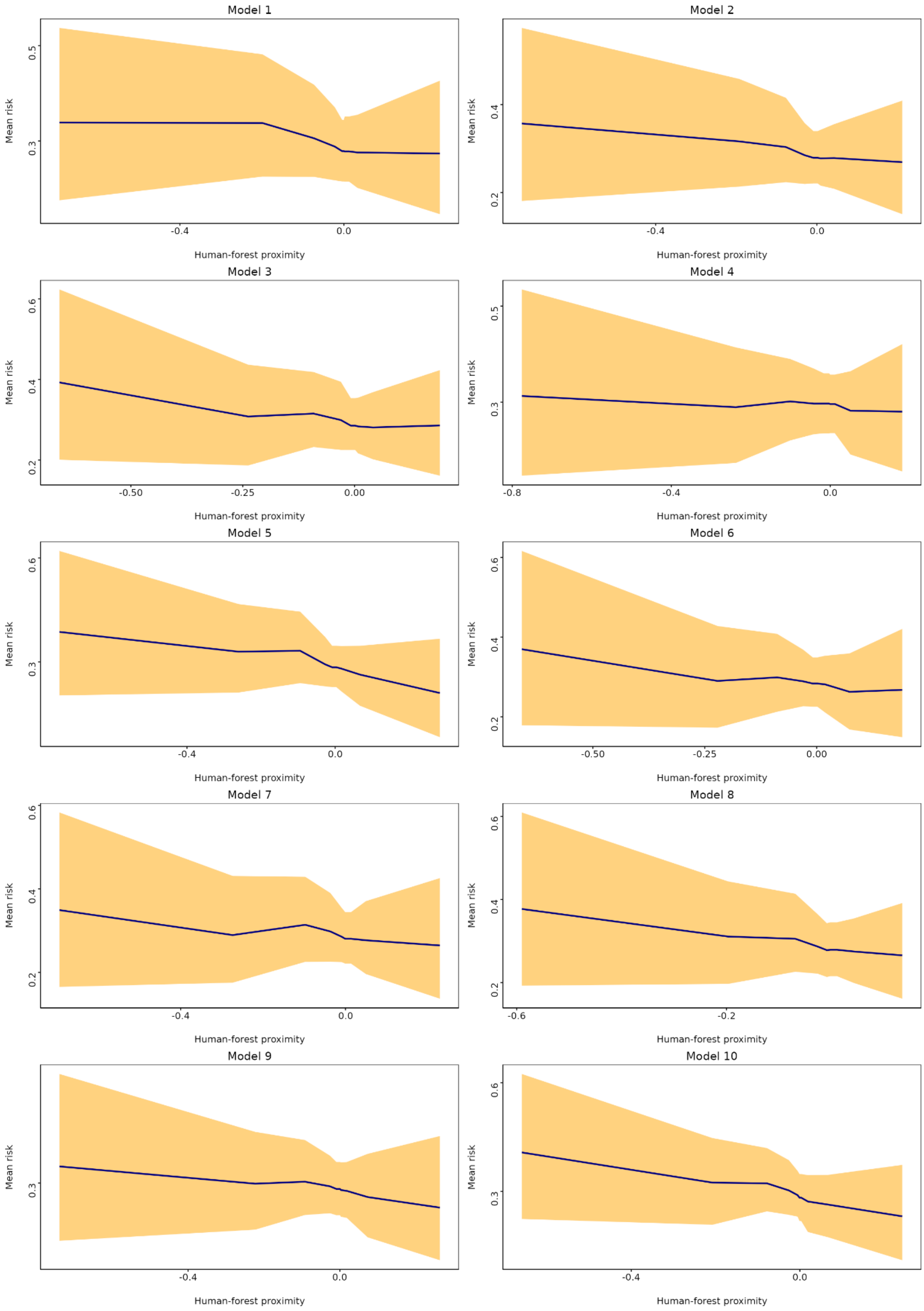

Model 1
Mean risk
0.5
0.3
-0.4
0.0
Human-forest proximity
Model 2
Mean risk
0.4
0.2
-0.4
0.0
Human-forest proximity
Model 3
Mean risk
0.6
0.4
0.2
-0.50
-0.25
0.00
Human-forest proximity
Model 4
Mean risk
0.5
0.3
-0.8
-0.4
0.0
Human-forest proximity
Model 5
Mean risk
0.6
0.3
-0.4
0.0
Human-forest proximity
Model 6
Mean risk
0.6
0.4
0.2
-0.50
-0.25
0.00
Human-forest proximity
Model 7
Mean risk
0.6
0.4
0.2
-0.4
0.0
Human-forest proximity
Model 8
Mean risk
0.6
0.4
0.2
-0.6
-0.2
Human-forest proximity
Model 9
Mean risk
0.3
-0.4
0.0
Human-forest proximity
Model 10
Mean risk
0.6
0.3
-0.4
0.0
Human-forest proximity

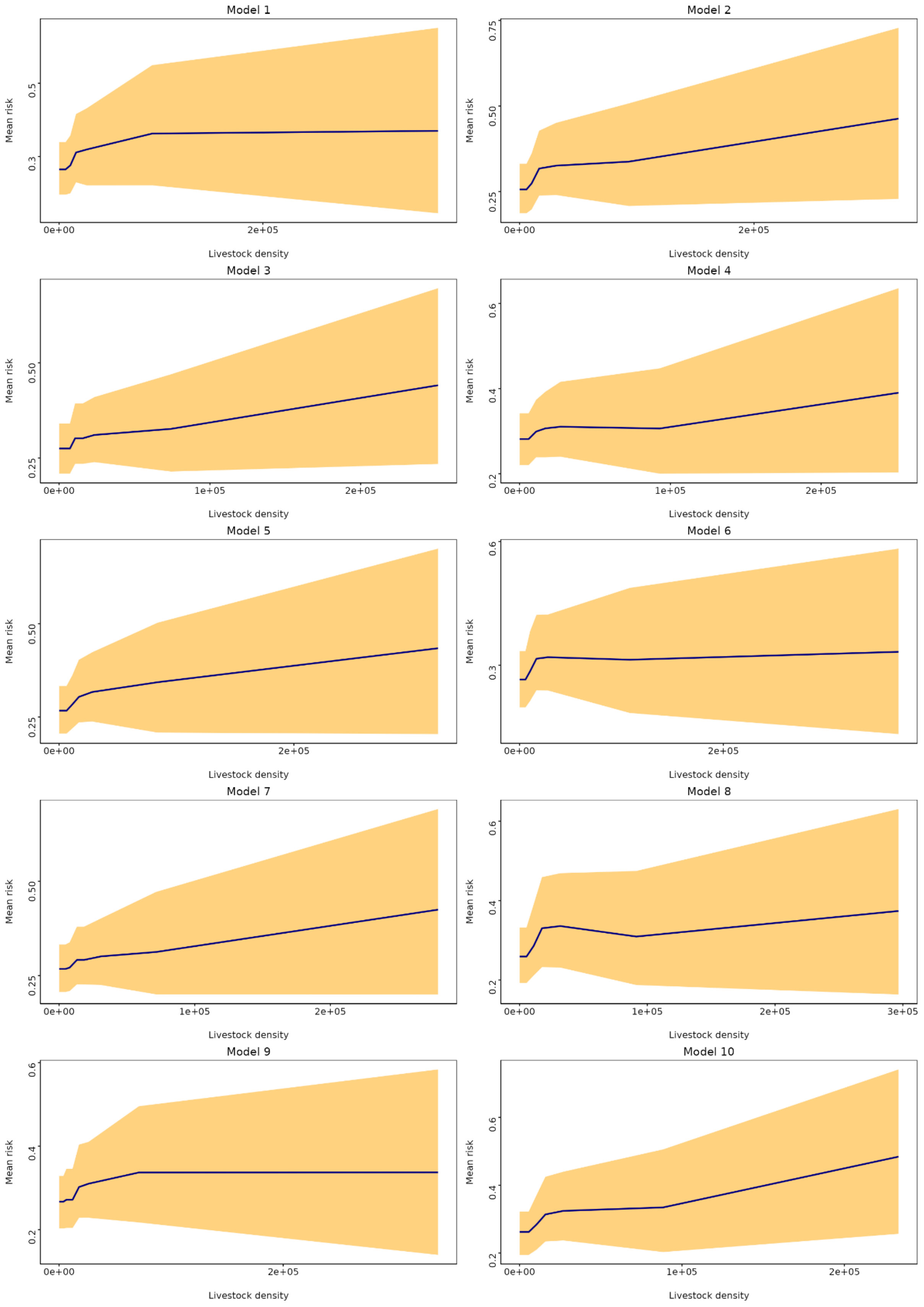

Model 1
Model 2
Model 3
Model 4
Model 5
Model 6
Model 7
Model 8
Model 9
Model 10
Mean risk
Livestock density
0.3
0.5
0.25
0.50
0.75
0.2
0.4
0.6
0e+00
1e+05
2e+05
3e+05

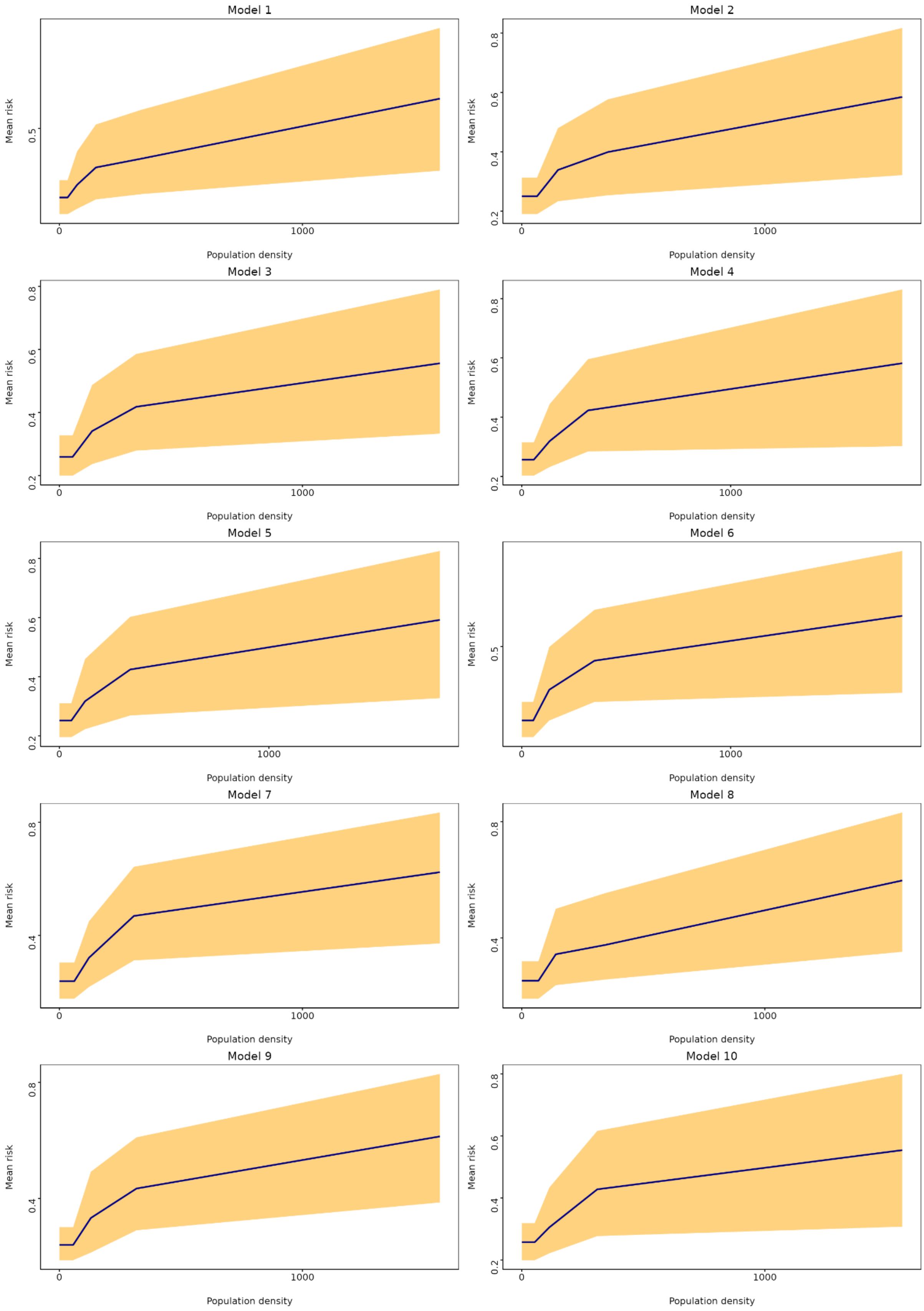
Model 1
Mean risk
0.5
0
1000
Population density
Model 2
Mean risk
0.2
0.4
0.6
0.8
0
1000
Population density
Model 3
Mean risk
0.2
0.4
0.6
0.8
0
1000
Population density
Model 4
Mean risk
0.2
0.4
0.6
0.8
0
1000
Population density
Model 5
Mean risk
0.2
0.4
0.6
0.8
0
1000
Population density
Model 6
Mean risk
0.5
0
1000
Population density
Model 7
Mean risk
0.4
0.8
0
1000
Population density
Model 8
Mean risk
0.4
0.8
0
1000
Population density
Model 9
Mean risk
0.4
0.8
0
1000
Population density
Model 10
Mean risk
0.2
0.4
0.6
0.8
0
1000
Population density

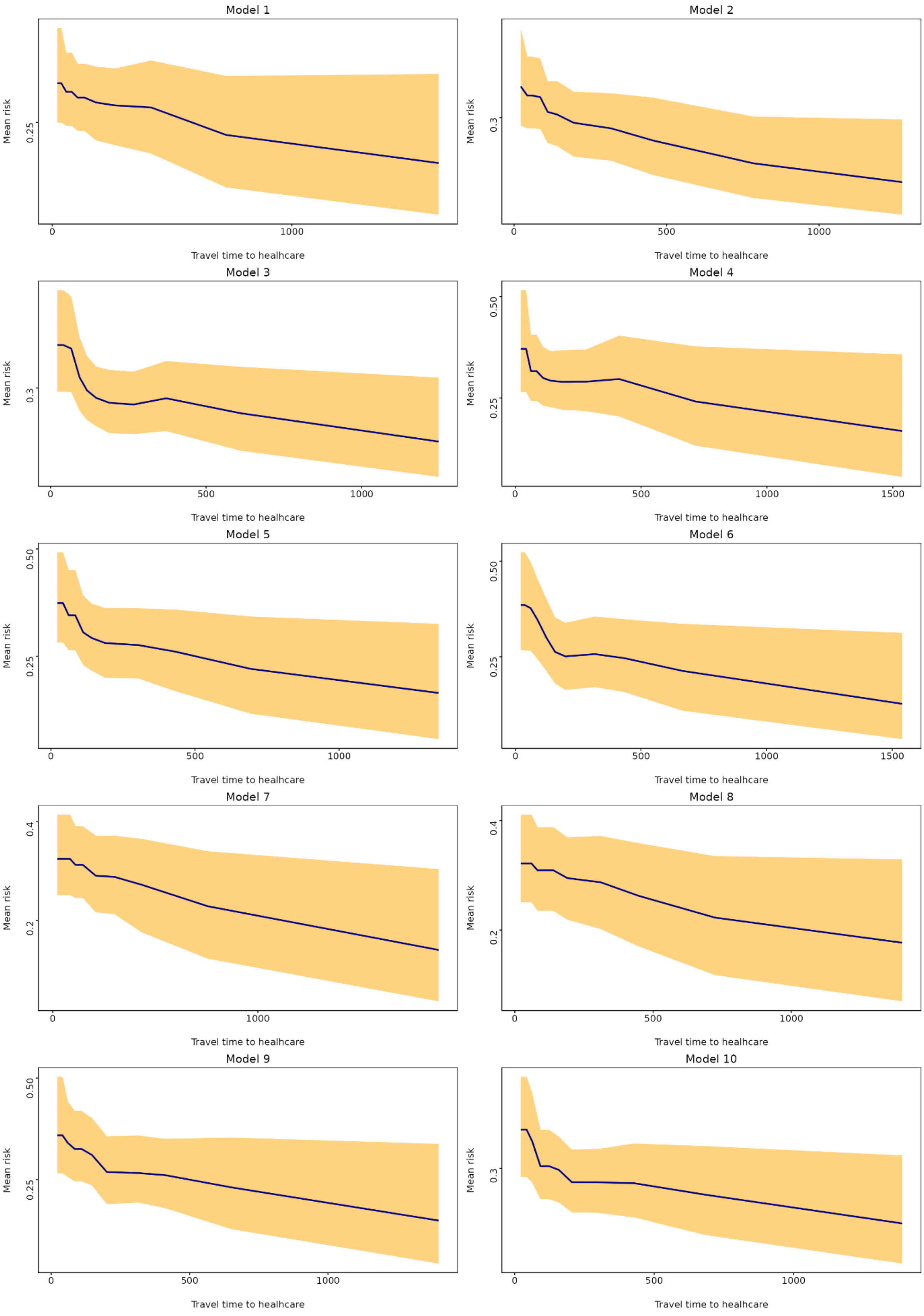

Fig. S5: Relationship between risk factors and the occurrence of diseases with epidemic and pandemic potential.

Relationship between risk factors and the occurrence of diseases with epidemic and pandemic potential as depicted by each model within the set of 10 BART models of model framework 1. The blue line represents the average risk, while the orange

area indicates the 95% confidence intervals derived from the quantiles of the posterior distribution. Marginal effects were quantified at specific quantiles—0.025, 0.1, 0.2, 0.3, 0.4, 0.5, 0.6, 0.7, 0.8, 0.9, and 0.975—to construct 95% confidence intervals.

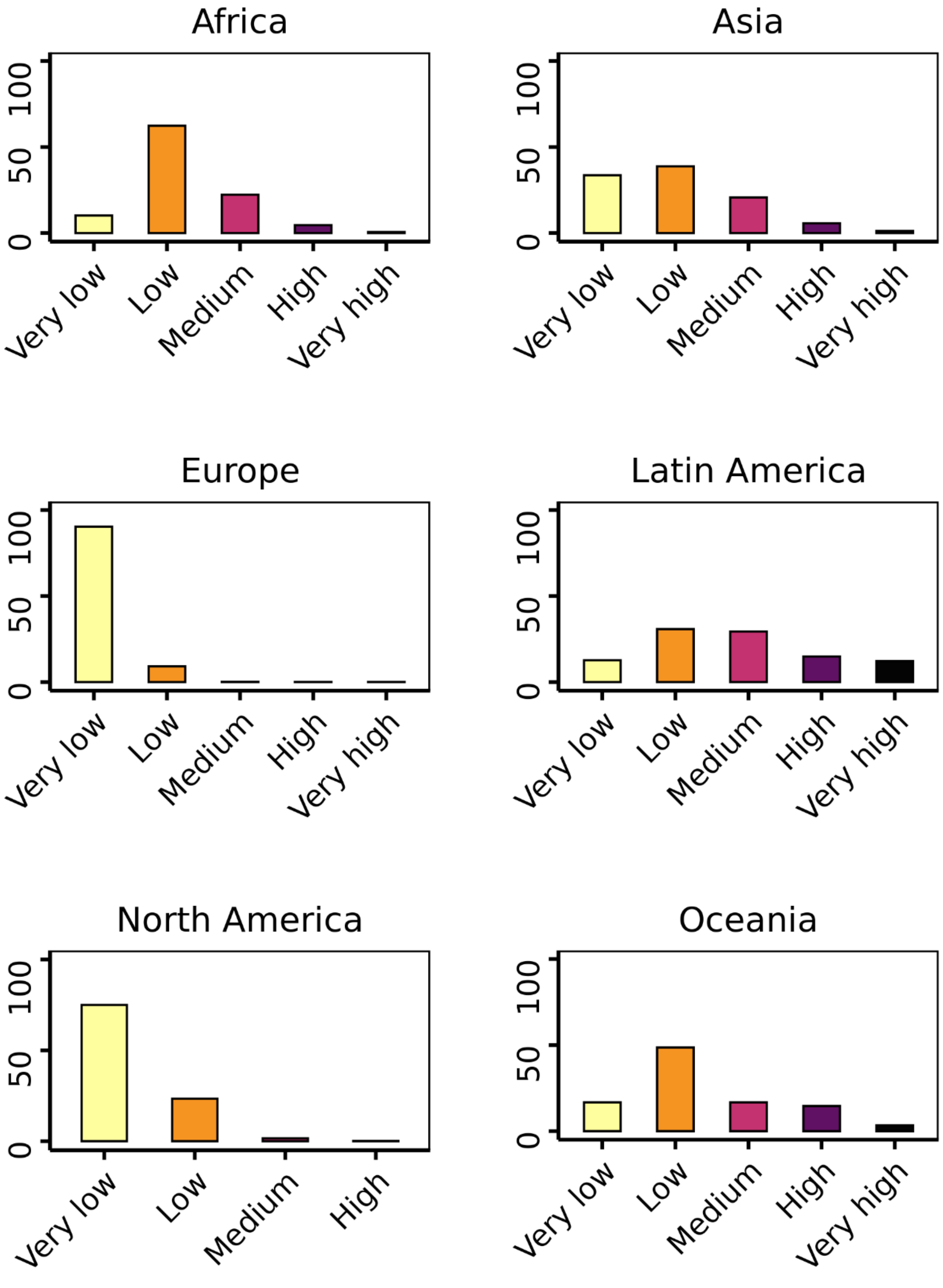

Fig. S6: Regional risk distribution.

Distribution of true risk categories by region. The x-axis represents the risk categories, while the y-axis represents the percentage of cells. The percentage of cells for each category was computed by dividing the total number of cells within each region.

**Category**	**Cells [%]**	**Region**
Very low	10.15	Africa
Low	62.36	Africa
Medium	22.31	Africa
High	4.58	Africa
Very high	0.6	Africa
Very low	33.58	Asia
Low	38.8	Asia
Medium	20.74	Asia
High	5.71	Asia
Very high	1.17	Asia
Very low	90.35	Europe
Low	9.23	Europe
Medium	0.23	Europe
High	0.11	Europe
Very high	0.09	Europe
Very low	12.7	Latin America
Low	30.76	Latin America
Medium	29.43	Latin America
High	14.94	Latin America
Very high	12.16	Latin America
Very low	74.97	North America
Low	23.36	North America
Medium	1.59	North America
High	0.08	North America
Very low	16.66	Oceania
Low	48.56	Oceania
Medium	16.72	Oceania
High	14.62	Oceania
Very high	3.44	Oceania

Table S1: Regional risk categories.

Percentage of cells within each true risk category by region. In the table percentages were rounded and may not total to 100%

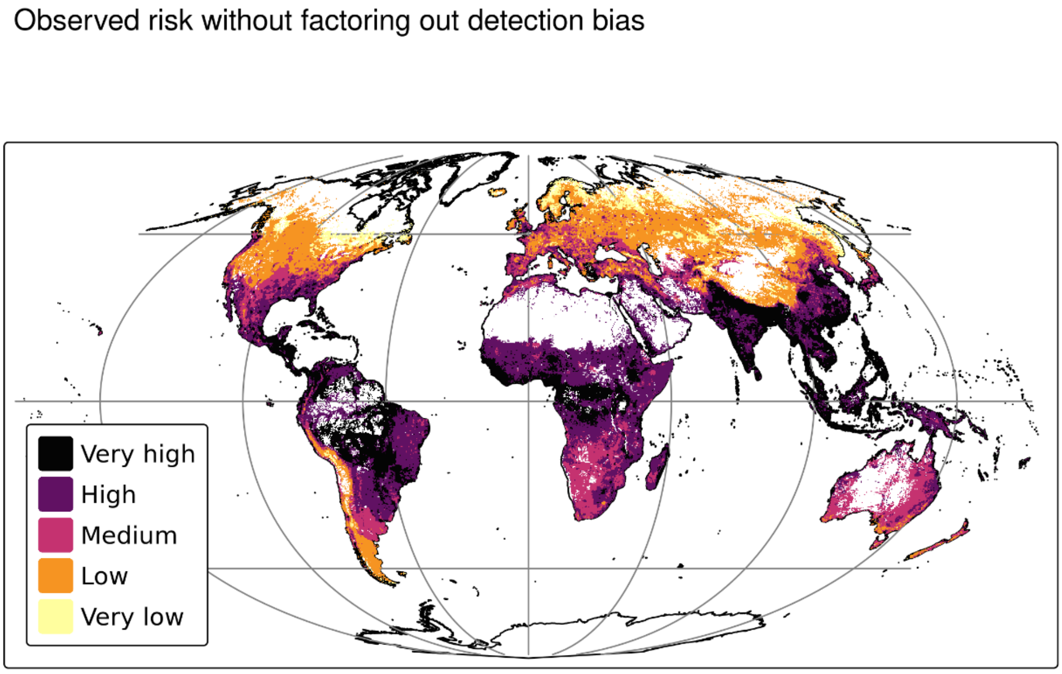

Fig. S7: Observed risk map.

Observed risk: this map displays the output of model framework 2, which was fitted using nine anthropogenic drivers without factoring out detection bias. The values represent the mean prediction across the 10 models in the ensemble, providing an average estimate of the probability of the observed risk. The four risk categories were defined based on Fisher's algorithm applied to the true risk values.

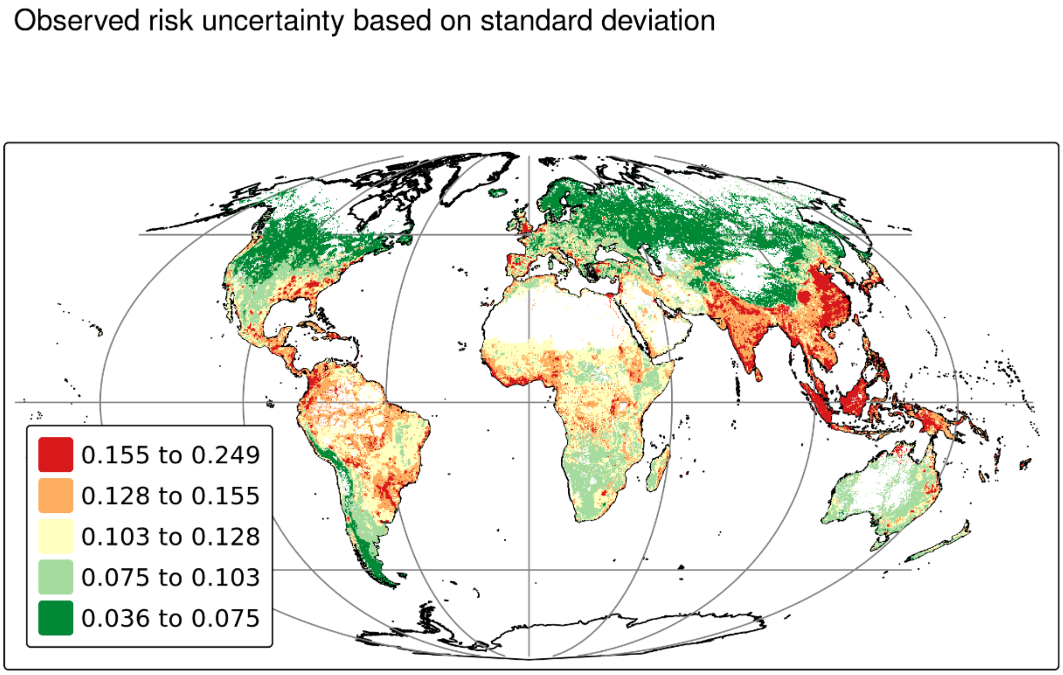

Fig. S8: Uncertainty predictions.

Uncertainty predictions for the observed risk of diseases with epidemic and pandemic potential in humans. This map displays the standard deviation of model framework 2, which was fitted using nine anthropogenic drivers without accounting for detection bias. The values represent the mean standard deviation across the 10 models in the ensemble, providing an estimate of the uncertainty of the observed risk. Colour categories are defined based on Fisher's algorithm

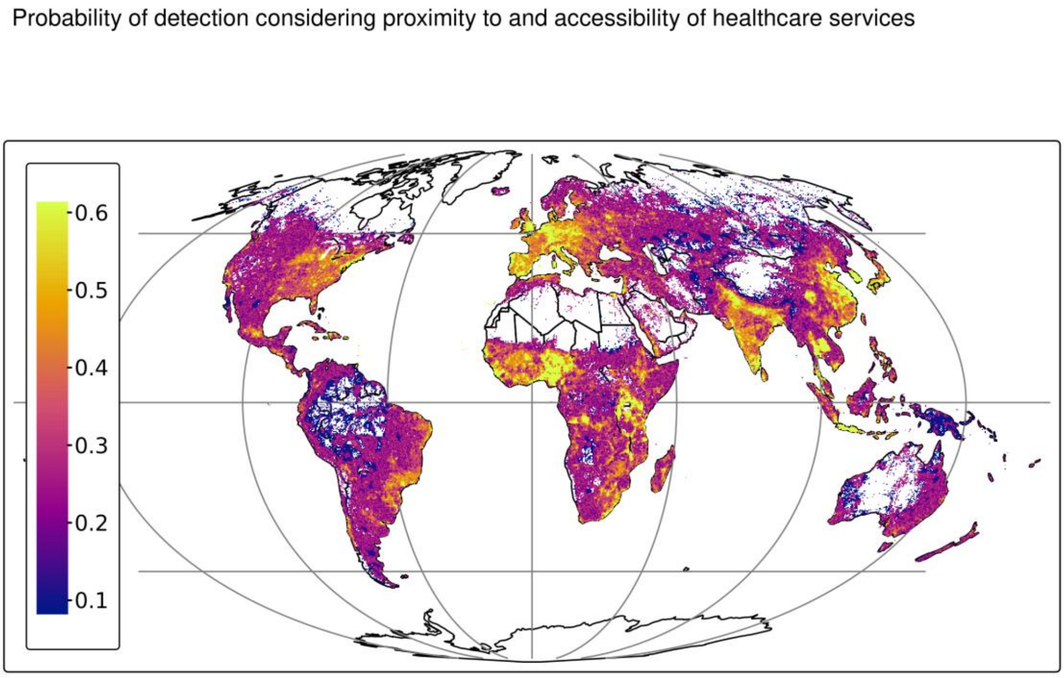

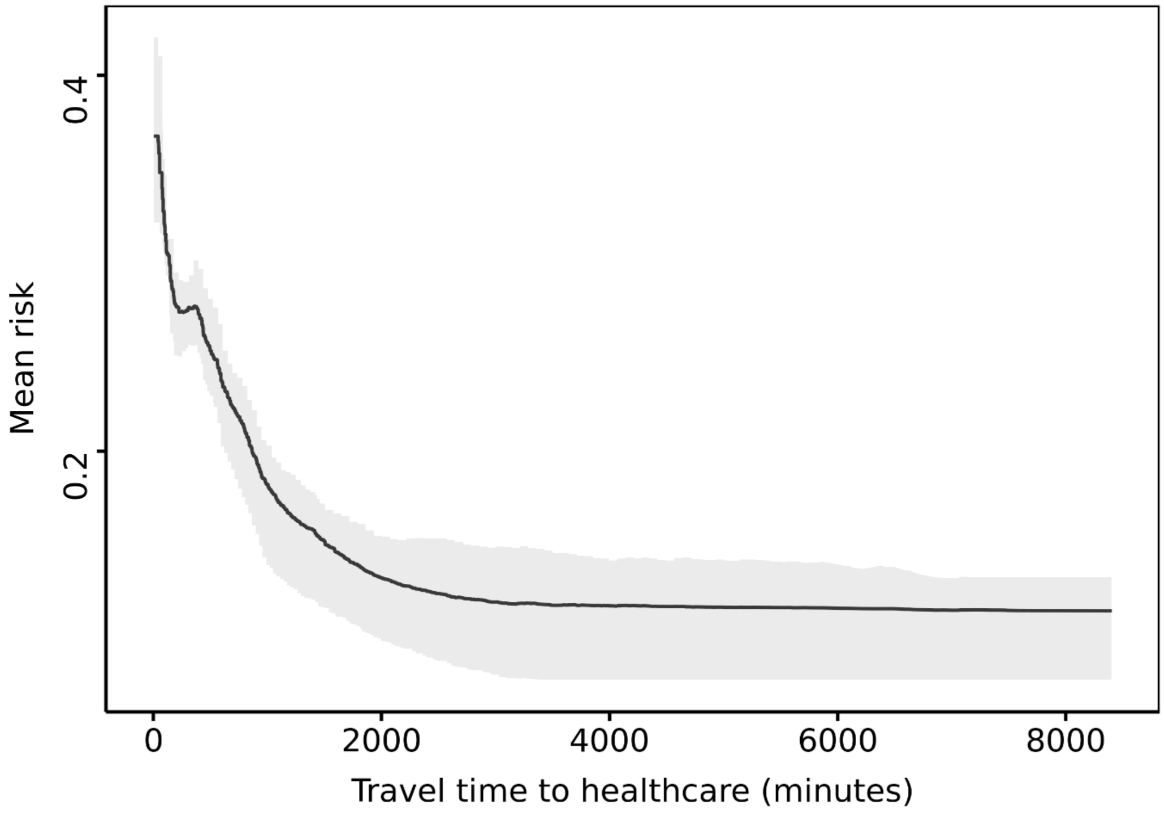

Fig. S9: Detection probability and marginal effects.

At the top: probability of detection. This map displays the output of model framework 3, which was fitted using travel time to healthcare facilities as the sole variable. The values represent the mean prediction across the 10 models in the ensemble,

providing an average estimate of the probability of detection. The colour scheme ranges from blue (lower probability) to yellow, highlighting areas where proximity to healthcare facilities enables effective detection and, consequently, a higher likelihood of outbreaks being reported. At the bottom: marginal effect of travel time to healthcare facilities calculated in model framework 1.

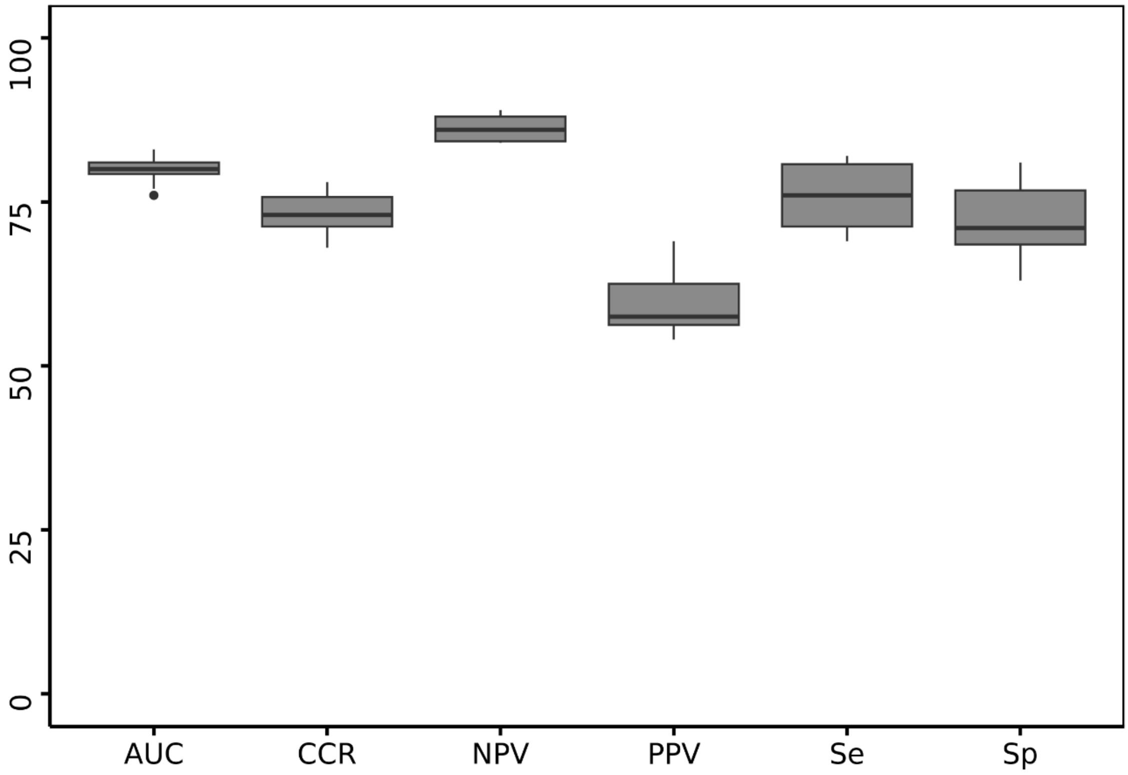

***Fig. S10: Model evaluation metrics*.**

Model framework 1: model evaluation metrics based on 3-fold cross-validation. The box plots show the average across 10 replicate models runs.

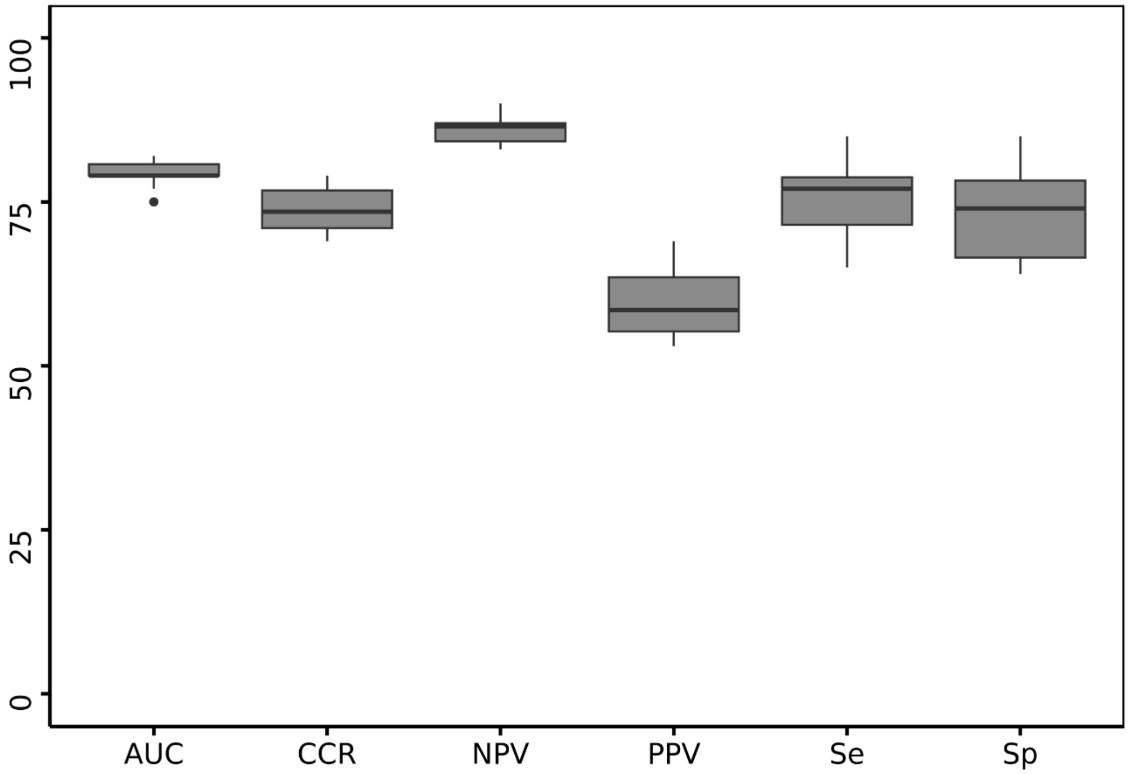

Fig. S11: Model evaluation metrics.

Model framework 2: model evaluation metrics based on 3-fold cross-validation. The box plots show the average across 10 replicate models runs.

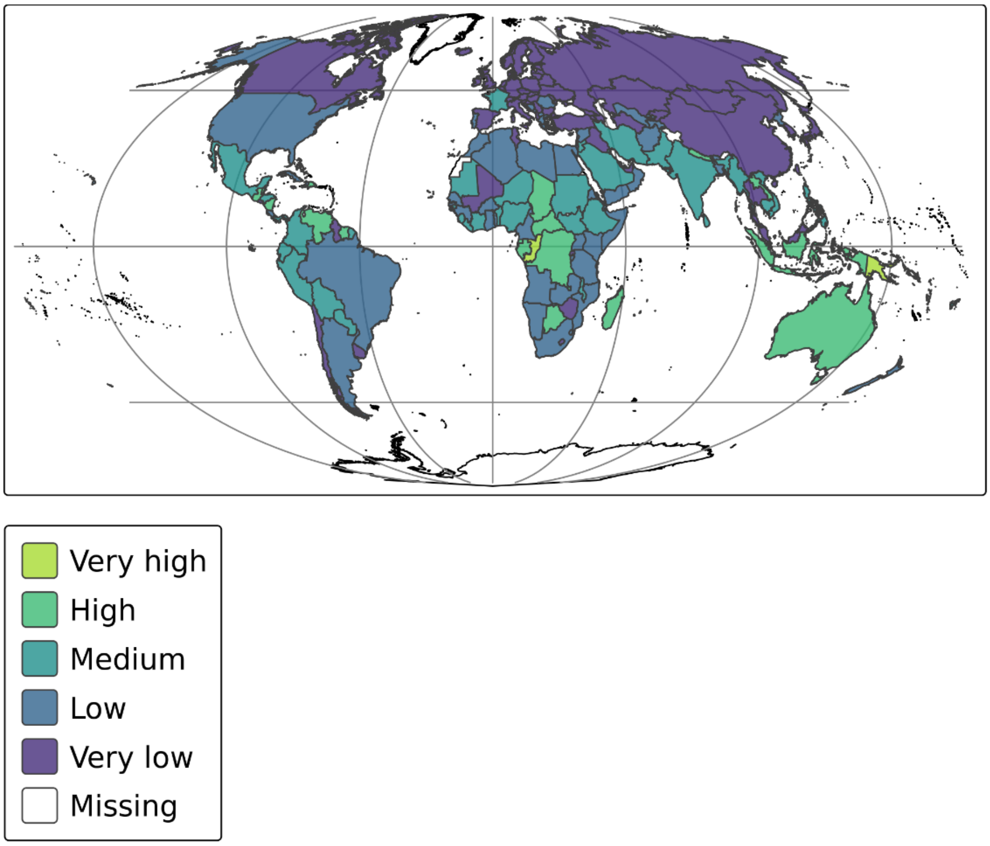

Fig. S12: Spatial distribution of epidemic risk (index).

Spatial distribution of epidemic risk (index)

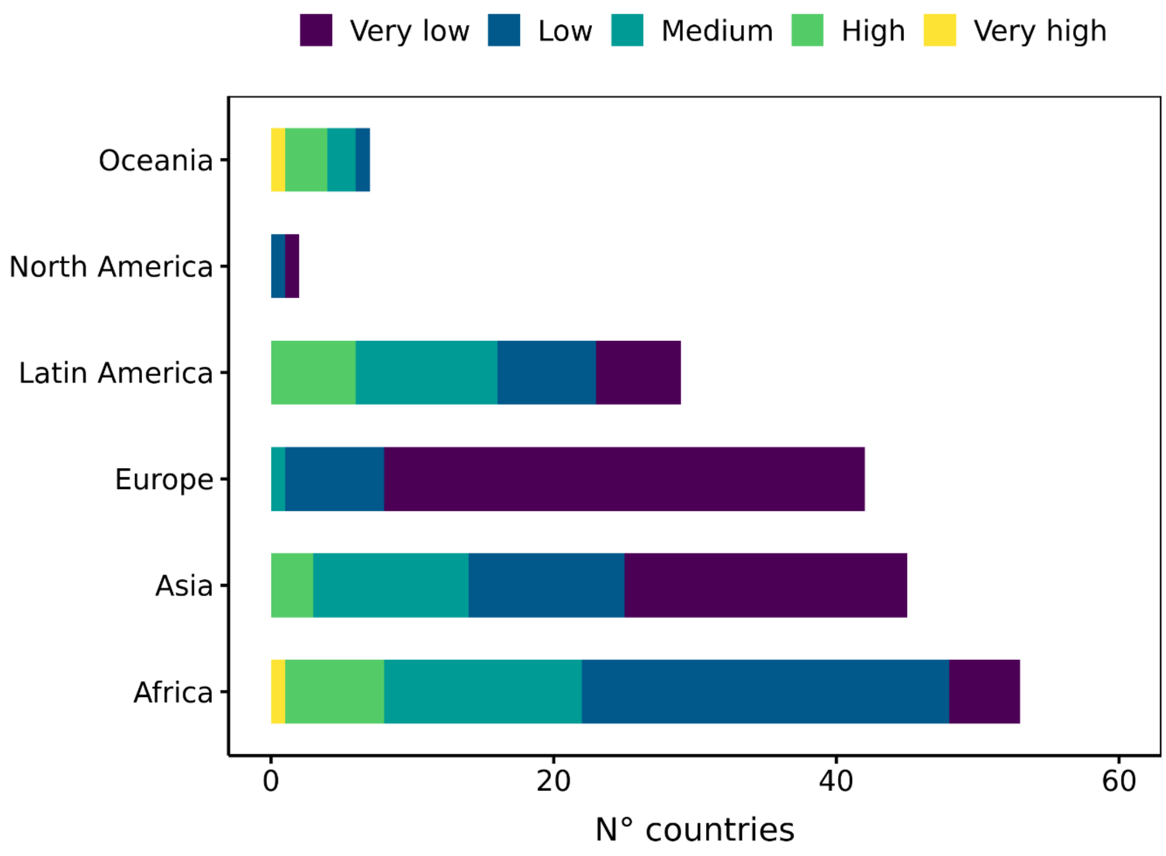

Fig. S13: Distribution of epidemic risk categories.

Distribution of epidemic risk (index) categories by region: number of countries per each epidemic risk (index) category. For visualization purposes, the number of countries at different levels of epidemic risk were computed by reclassifying the values into five classes, very low ($x < 0.044$), low ($0.044 \leq x < 0.115$), medium ($0.115 \leq x < 0.231$), high ($0.231 \leq x < 0.418$), and very high ($0.418 \leq x$).

Rank	Country	Max risk	IHR C3	Epidemic risk (index)
1	Papua New Guinea	0.645	0.200	0.516
2	Congo	0.668	0.267	0.490
3	Vanuatu	0.433	0.200	0.347
4	Australia	0.644	0.467	0.344
5	Laos	0.553	0.400	0.332
6	Central African Republic	0.386	0.200	0.308
7	Solomon Islands	0.479	0.400	0.288
8	Madagascar	0.349	0.200	0.279
9	Nepal	0.347	0.200	0.277
10	Gabon	0.512	0.467	0.273
11	Indonesia	1.000	0.733	0.267
12	Belize	0.373	0.300	0.261
13	Democratic Republic of the Congo	0.635	0.600	0.254
14	Guatemala	0.474	0.467	0.253
15	Bahamas	0.533	0.533	0.249
16	Chad	0.372	0.333	0.248

17	Botswana	0.305	0.200	0.244
18	Nicaragua	0.721	0.667	0.240
19	Suriname	0.718	0.667	0.239
20	Equatorial Guinea	0.296	0.200	0.237
21	Venezuela	0.878	0.733	0.234
22	Dominican Republic	0.361	0.400	0.217
23	South Sudan	0.313	0.333	0.209
24	Paraguay	0.433	0.533	0.202
25	Saudi Arabia	0.421	0.533	0.196
26	Peru	0.692	0.733	0.185
27	Guinea-Bissau	0.342	0.467	0.182
28	Trinidad and Tobago	0.225	0.200	0.180
29	Cambodia	0.450	0.600	0.180
30	Samoa	0.223	0.200	0.178
31	Sri Lanka	0.220	0.200	0.176
32	Myanmar/Burma	0.870	0.800	0.174
33	Niger	0.288	0.400	0.173
34	Mexico	0.510	0.667	0.170
35	Vietnam	0.424	0.600	0.170
36	India	0.418	0.600	0.167
37	Bolivia	0.809	0.800	0.162
38	Colombia	0.792	0.800	0.158
39	Nigeria	0.387	0.600	0.155
40	Philippines	0.507	0.700	0.152
41	Honduras	0.758	0.800	0.152
42	Pakistan	0.379	0.600	0.151
43	Ethiopia	0.323	0.533	0.151
44	France	0.743	0.800	0.149
45	Côte d'Ivoire	0.371	0.600	0.148
46	São Tomé and Príncipe	0.222	0.333	0.148
47	Panama	0.724	0.800	0.145
48	Mauritania	0.349	0.600	0.140
49	Sierra Leone	0.229	0.400	0.137
50	Benin	0.170	0.200	0.136
51	Malawi	0.284	0.533	0.132
52	Comoros	0.276	0.533	0.129
53	Liberia	0.374	0.667	0.125
54	Iran	0.407	0.700	0.122
55	Kuwait	0.262	0.533	0.122
56	Fiji	0.305	0.600	0.122
57	Sudan	0.454	0.733	0.121
58	Jordan	0.296	0.600	0.118
59	Ecuador	0.587	0.800	0.117
60	Uganda	0.243	0.533	0.114
61	Timor-Leste	0.149	0.267	0.109
62	Guinea	0.316	0.667	0.105

63	Libya	0.370	0.733	0.099
64	Mauritius	0.123	0.200	0.099
65	Syria	0.290	0.667	0.097
66	Saint Vincent and The Grenadines	0.160	0.400	0.096
67	Zambia	0.158	0.400	0.095
68	Kenya	0.236	0.600	0.094
69	Uzbekistan	0.281	0.667	0.094
70	Costa Rica	0.351	0.733	0.094
71	Bosnia and Herzegovina	0.115	0.200	0.092
72	United Republic of Tanzania	0.229	0.600	0.091
73	Romania	0.111	0.200	0.089
74	Cameroon	0.332	0.733	0.089
75	Somalia	0.221	0.600	0.088
76	Burundi	0.164	0.467	0.087
77	Bangladesh	0.430	0.800	0.086
78	Eritrea	0.215	0.600	0.086
79	Burkina Faso	0.183	0.533	0.085
80	Djibouti	0.142	0.400	0.085
81	Egypt	0.418	0.800	0.084
82	Algeria	0.410	0.800	0.082
83	Barbados	0.133	0.400	0.080
84	Senegal	0.199	0.600	0.080
85	Gambia	0.097	0.200	0.078
86	Togo	0.165	0.533	0.077
87	Ghana	0.186	0.600	0.074
88	Bhutan	0.372	0.800	0.074
89	Argentina	0.278	0.733	0.074
90	Tajikistan	0.243	0.700	0.073
91	Greece	0.180	0.600	0.072
92	Morocco	0.348	0.800	0.070
93	Afghanistan	0.343	0.800	0.069
94	Namibia	0.257	0.733	0.068
95	Yemen	0.328	0.800	0.066
96	South Africa	0.245	0.733	0.065
97	Cuba	0.305	0.800	0.061
98	Eswatini	0.091	0.333	0.061
99	Haiti	0.151	0.600	0.060
100	United States	0.286	0.800	0.057
101	Mozambique	0.212	0.733	0.056
102	New Zealand	0.141	0.600	0.056
103	North Korea	0.280	0.800	0.056
104	Oman	0.404	0.867	0.054
105	San Marino	0.067	0.200	0.053
106	North Macedonia	0.113	0.533	0.053
107	Angola	0.396	0.867	0.053
108	Brazil	0.779	0.933	0.052

109	Montenegro	0.110	0.533	0.052
110	Cape Verde	0.089	0.467	0.048
111	Portugal	0.078	0.400	0.047
112	Cyprus	0.171	0.733	0.046
113	Lesotho	0.069	0.400	0.041
114	Mali	0.201	0.800	0.040
115	Kazakhstan	0.198	0.800	0.040
116	Zimbabwe	0.148	0.733	0.039
117	Uruguay	0.195	0.800	0.039
118	Rwanda	0.139	0.733	0.037
119	Israel	0.175	0.800	0.035
120	Jamaica	0.257	0.867	0.034
121	Malaysia	0.506	0.933	0.034
122	Moldova	0.079	0.600	0.032
123	Ukraine	0.116	0.733	0.031
124	Albania	0.151	0.800	0.030
125	Azerbaijan	0.147	0.800	0.029
126	Singapore	0.146	0.800	0.029
127	Lebanon	0.105	0.733	0.028
128	Chile	0.205	0.867	0.027
129	Serbia	0.130	0.800	0.026
130	Slovakia	0.055	0.533	0.026
131	Türkiye	0.174	0.867	0.023
132	Spain	0.115	0.800	0.023
133	Saint Lucia	0.115	0.800	0.023
134	Georgia	0.110	0.800	0.022
135	Ireland	0.055	0.600	0.022
136	Turkmenistan	0.320	0.933	0.021
137	Kyrgyzstan	0.101	0.800	0.020
138	Bulgaria	0.091	0.800	0.018
139	Hungary	0.062	0.733	0.017
140	Estonia	0.082	0.800	0.016
141	Bahrain	0.159	0.900	0.016
142	Tunisia	0.232	0.933	0.015
143	Croatia	0.076	0.800	0.015
144	Andorra	0.038	0.600	0.015
145	Latvia	0.072	0.800	0.014
146	Iceland	0.065	0.800	0.013
147	Lithuania	0.059	0.800	0.012
148	Belarus	0.056	0.800	0.011
149	Czechia	0.047	0.800	0.009
150	Slovenia	0.047	0.900	0.005
151	Liechtenstein	0.017	0.800	0.003
152	Luxembourg	0.020	0.933	0.001
153	United Arab Emirates	0.398	1.000	0.000
154	Armenia	0.083	1.000	0.000

155	Austria	0.053	1.000	0.000
156	Belgium	0.066	1.000	0.000
157	Canada	0.165	1.000	0.000
158	Switzerland	0.036	1.000	0.000
159	China	0.346	1.000	0.000
160	Denmark	0.043	1.000	0.000
161	Germany	0.070	1.000	0.000
162	Finland	0.073	1.000	0.000
163	Guyana	0.724	1.000	0.000
164	Iraq	0.373	1.000	0.000
165	Italy	0.074	1.000	0.000
166	Japan	0.115	1.000	0.000
167	South Korea	0.092	1.000	0.000
168	Mongolia	0.192	1.000	0.000
169	Monaco	0.071	1.000	0.000
170	Netherlands	0.066	1.000	0.000
171	Norway	0.076	1.000	0.000
172	Russian Federation	0.158	1.000	0.000
173	Poland	0.054	1.000	0.000
174	Qatar	0.208	1.000	0.000
175	Sweden	0.052	1.000	0.000
176	El Salvador	0.225	1.000	0.000
177	Thailand	0.375	1.000	0.000
178	United Kingdom	0.090	1.000	0.000

Table S2: Country-level risk data.

Maximum risk of an outbreak, epidemic risk (index) and IHR C3 by country.

Supplementary text

Details on host species and transmission routes

In this study, we focus on Crimean-Congo haemorrhagic fever (CCHF), Ebola virus disease, Lassa fever, Middle East respiratory syndrome (MERS), Severe Acute Respiratory Syndrome (SARS), Marburg virus disease (MVD), Nipah virus disease (NiV), Rift Valley Fever (RVF), and Zika. These diseases affect a wide range of wild and domestic animals (*62*–*67*). In general, human-to-human transmission of these pathogens is occasional or limited to specific settings (*62*, *65*, *66*, *68*, *69*). In particular, hares, cattle, horses, goats, and sheep play a significant role in the epidemiology of CCHF (*62*). This disease is primarily transmitted to humans and animals through bites from *Ixodid* ticks, predominantly of the *Hyalomma* genus, or through direct contact with the blood of infected animals. Individuals working in agro-pastoral or animal husbandry fields, who are in contact with fresh flesh and blood of animals, are at the highest risk of infection (*16*, *62*). However, human-to-human transmission can occur through contact with the blood and other body fluids of viraemic patients, but this is limited to hospital settings (*70*). For Ebola, the precise identity of the natural reservoir remains elusive. Some hypotheses suggest that a small mammal, which may not exhibit prominent symptoms, could serve as the reservoir (*71*). Others propose bats as the likely reservoir species(*72*). Duikers, non-human primates, felids, canids, swine, antelopes, porcupines, and rodents are among the animals identified as potential intermediate or incidental hosts for the virus (*73*). These species may play roles in the transmission dynamics of the virus in affected ecosystems (*73*). During an outbreak, the initial human case is

typically linked to contact with an infected animal, such as handling bushmeat or exposure to bats, rodents, or primates, whether alive or deceased (*74*). Subsequent human-to-human transmission can occur through direct contact with the blood or body fluids of an infected person, or via contaminated objects, including materials that have come into contact with these fluids from someone sick with or deceased from Ebola (*74*). For Lassa fever, nosocomial transmission is possible (e.g. through a contaminated needle) but the majority of infections in humans occur via direct or indirect contact with infected rodent excreta (*64*). The transmission of MERS primarily involves repeated spillover events from animals to humans, often through direct or indirect contact with infected camels or camel-derived products, such as raw camel milk or camel urine. Additionally, secondary human-to-human transmission frequently occurs in healthcare settings (*68*). With regard to SARS, it is thought that the virus was transmitted from bats to humans via palm civets (*75*). The outbreak of SARS (2002-2003) spread across some countries, but it was largely contained due to the relatively inefficient human-to-human transmission, compared to COVID-19, for example (*67*). There were also cases linked to restaurant environments suggesting interspecies transfer from the presumed palm civet reservoir, rather than ongoing transmission of SARS-CoV among humans (*76*). For MVD, almost all outbreaks were linked to humans entering bat-infested caves, including cave visitors and miners (*69*). Like the above mentioned diseases, human-to-human transmission generally occurs through direct contact with the bodily fluids of infected individuals (*69*). Of note is the fact that the WHO emphasizes that, due to their demonstrated experimental susceptibility, pigs should be regarded as potential amplifier hosts for the virus until proven otherwise (*77*). Fruit bats are the natural reservoir hosts of NiV, with transmissions to humans often involving an intermediate host (i.e. pigs), which can amplify and spread the virus (*65*). Additionally, many small animals have been shown to support viral replication when experimentally infected with NiV (*65*). For RFV, there is no documented direct horizontal human-to-human transmission and the transmission occurs contact with blood, body fluids, or tissues and organs of infected animals and aborted animal foetuses, and through mosquito bites (*66*). Lastly, Zika virus transmission primarily occurs through infected female mosquitoes of the *Aedes* genus, with the infection being widespread among various animal hosts and vectors(*63*). Antibodies against the virus have been found in ducks, goats, cows, horses, bats, and carabaos (water buffaloes from the Philippines), suggesting extensive virus circulation among domestic and wild animals (*63*). Unlike the diseases mentioned earlier, in urban areas, humans may act as amplification hosts for Zika, and alternative transmission routes are possible, such as sexual and mother-to-fetus (*63*).

Role of anthropogenic factors in the risk of outbreaks of diseases with epidemic and pandemic potential

Anthropogenic changes directly and indirectly affect the risk of diseases like RVF, CCHF, and Zika, which are vector-borne. With a changing climate, the potential for expanded transmission risk of those diseases due to increased thermal suitability was anticipated (*16*, *17*). Additionally, heavy rainfall and flooding have been linked to their emergence (*18*). Indirect climate effect on vector-borne diseases includes changing the migration pattern of ground feeding birds carrying ticks infected with CCHF and as well as impacting on their reservoir abundance (hares), which may increase through milder winters and enhanced heavy rainfall (*16*).

Non-vector-borne diseases like Ebola are influenced by climate, with outbreaks linked to the end of African rainy season potentially increasing human contact with infected reservoirs like bats during periods of fruit abundance(*20*). Indeed, changes in climate lead to unusual levels of contact between reservoir hosts and humans in a variety of ways. Heavy rainfall may cause rodent populations to surge, triggering more outbreaks of Lassa fever, particularly in poorly-built homes or areas with low hygiene standards(*21*). Coastal flooding displacing people to higher grounds also raises the danger of Lassa fever unless proper housing and rodent management are ensured (*21*).

Most of the WHO priority diseases have their reservoir in bats, but other wildlife species are susceptible hosts (*20*, *65*, *76*, *77*). Thus, forest fragmentation and deforestation, often due to logging activities,

facilitate human-wildlife interaction (*20*). Seasonal labour influx in forests, attracted by oil palm fruit harvests, further increases human presence. Agricultural expansion and urban growth reduce the buffer between cities and forests, prompting deforestation and greater human access to forest areas (*20*, *65*). Such habitat loss alters wildlife foraging patterns, often leading to reliance on anthropogenic food sources, and intensifies contacts among bats, humans, and livestock, potentially increasing disease spillover risk (*78*).

REFERENCES AND NOTES

1. K. E. Jones, N. G. Patel, M. A. Levy, A. Storeygard, D. Balk, J. L. Gittleman, P. Daszak, Global trends in emerging infectious diseases. *Nature* **451**, 990–993 (2008).

2. A. J. Meadows, N. Stephenson, N. K. Madhav, B. Oppenheim, Historical trends demonstrate a pattern of increasingly frequent and severe spillover events of high-consequence zoonotic viruses. *BMJ Glob. Health* **8**, e012026 (2023).

3. A. El-Sayed, M. Kamel, Climatic changes and their role in emergence and re-emergence of diseases. *Environ. Sci. Pollut. Res.* **27**, 22336–22352 (2020).

4. N. Nova, T. S. Athni, M. L. Childs, L. Mandle, E. A. Mordecai, Global change and emerging infectious diseases. *Infect. Dis.* **14**, 333–354 (2022).

5. H. Bartlett, M. A. Holmes, S. O. Petrovan, D. R. Williams, J. L. N. Wood, A. Balmford, Understanding the relative risks of zoonosis emergence under contrasting approaches to meeting livestock product demand. *R. Soc. Open Sci.* **9**, 211573 (2022).

6. D. Nepstad, D. McGrath, C. Stickler, A. Alencar, A. Azevedo, B. Swette, T. Bezerra, M. DiGiano, J. Shimada, R. Seroa Da Motta, E. Armijo, L. Castello, P. Brando, M. C. Hansen, M. McGrath-Horn, O. Carvalho, L. Hess, Slowing Amazon deforestation through public policy and interventions in beef and soy supply chains. *Science* **344**, 1118–1123 (2014).

7. IPBES, *Global Assessment Report on Biodiversity and Ecosystem Services of the Intergovernmental Science-Policy Platform on Biodiversity and Ecosystem Services* (Zenodo, 2019); https://doi.org/10.5281/zenodo.3831673.

8. World Health Organization, Convention on Biological Diversity, *Connecting Global Priorities: Biodiversity and Human Health: A State of Knowledge Review* (World Health Organization, 2015); https://iris.who.int/handle/10665/174012.

9. J. P. Messina, D. M. Pigott, N. Golding, K. A. Duda, J. S. Brownstein, D. J. Weiss, H. Gibson, T. P. Robinson, M. Gilbert, G. R. William Wint, P. A. Nuttall, P. W. Gething, M. F. Myers, D.

B. George, S. I. Hay, The global distribution of Crimean-Congo hemorrhagic fever. *Trans. R. Soc. Trop. Med. Hyg.* **109**, 503–513 (2015).

10. J. P. Messina, M. U. Kraemer, O. J. Brady, D. M. Pigott, F. M. Shearer, D. J. Weiss, N. Golding, C. W. Ruktanonchai, P. W. Gething, E. Cohn, J. S. Brownstein, K. Khan, A. J. Tatem, T. Jaenisch, C. J. Murray, F. Marinho, T. W. Scott, S. I. Hay, Mapping global environmental suitability for Zika virus. *eLife* **5**, e15272 (2016).

11. P. Van De Vuurst, L. E. Escobar, Climate change and infectious disease: A review of evidence and research trends. *Infect. Dis. Poverty* **12**, 51 (2023).

12. R. Gibb, S. J. Ryan, D. Pigott, M. D. P. Fernandez, R. L. Muylaert, G. F. Albery, D. J. Becker, J. K. Blackburn, H. Caceres-Escobar, M. Celone, E. A. Eskew, H. K. Frank, B. A. Han, E. N. Hulland, K. E. Jones, R. Katz, A. Kucharski, D. Limmathurotsakul, C. A. Lippi, J. Longbottom, J. F. Martinez, J. P. Messina, E. O. Nsoesie, D. W. Redding, D. Romero-Alvarez, B. V. Schmid, S. N. Seifert, A. Sinchi, C. H. Trisos, M. Wille, C. J. Carlson, The anthropogenic fingerprint on emerging infectious diseases. medRxiv 2024.05.22.24307684 [Preprint] (2024). https://doi.org/10.1101/2024.05.22.24307684.

13. T. Allen, K. A. Murray, C. Zambrana-Torrelio, S. S. Morse, C. Rondinini, M. Di Marco, N. Breit, K. J. Olival, P. Daszak, Global hotspots and correlates of emerging zoonotic diseases. *Nat. Commun.* **8**, 1124 (2017).

14. WHO, "Blueprint for R&D preparedness and response to public health emergencies due to highly infectious pathogens," 8–9 December 2015; https://cdn.who.int/media/docs/default-source/blue-print/2015-workshop-on-prioritization-of-pathogens.pdf?sfvrsn=50b6ddaa_2.

15. WHO, "WHO to identify pathogens that could cause future outbreaks and pandemics," 21 November 2022; www.who.int/news/item/21-11-2022-who-to-identify-pathogens-that-could-cause-future-outbreaks-and-pandemics.

16. A. Fanelli, D. Buonavoglia, Risk of Crimean Congo haemorrhagic fever virus (CCHFV) introduction and spread in CCHF-free countries in southern and Western Europe: A semi-quantitative risk assessment. *One Health* **13**, 100290 (2021).

17. S. J. Ryan, C. J. Carlson, B. Tesla, M. H. Bonds, C. N. Ngonghala, E. A. Mordecai, L. R. Johnson, C. C. Murdock, Warming temperatures could expose more than 1.3 billion new people to Zika virus risk by 2050. *Glob. Change Biol.* **27**, 84–93 (2021).

18. V. Martin, V. Chevalier, P. Ceccato, A. Anyamba, L. D. Simone, J. Lubroth, S. de La Rocque, J. Domenech, The impact of climate change on the epidemiology and control of Rift Valley fever. *Rev. Sci. Tech.* **27**, 413–426 (2008).

19. T. Alcayna, I. Fletcher, R. Gibb, L. Tremblay, S. Funk, B. Rao, R. Lowe, Climate-sensitive disease outbreaks in the aftermath of extreme climatic events: A scoping review. *One Earth* **5**, 336–350 (2022).

20. European Food Safety Authority (EFSA), Drivers for occasional spillover event of Ebola virus. *EFSA J.* **13**, 4161 (2015).

21. J. C. Clegg, Influence of climate change on the incidence and impact of arenavirus diseases: A speculative assessment. *Clin. Microbiol. Infect.* **15**, 504–509 (2009).

22. E. Mordecai, J. Caldwell, M. Grossman, C. Lippi, L. Johnson, M. Neira, J. Rohr, S. Ryan, V. Savage, M. Shocket, R. Sippy, A. Stewart Ibarra, M. Thomas, O. Villena, Thermal biology of mosquito-borne disease. *Ecol. Lett.* **10**, 1690–1708 (2019).

23. J. Rocklöv, R. Dubrow, Climate change: An enduring challenge for vector-borne disease prevention and control. *Nat. Immunol.* **21**, 479–483 (2020).

24. NASA, "Part 2: Selected Findings of the IPCC Special Report on Global Warming," 19 June 2019; https://climate.nasa.gov/news/2865/a-degree-of-concern-why-global-temperatures-matter/.

25. J. E. Coalson, E. J. Anderson, E. M. Santos, V. Madera Garcia, J. K. Romine, J. K. Luzingu, B. Dominguez, D. M. Richard, A. C. Little, M. H. Hayden, K. C. Ernst, The complex epidemiological relationship between flooding events and human outbreaks of mosquito-borne diseases: A scoping review. *Environ. Health Perspect.* **129**, 096002 (2021).

26. A. Anyamba, K. J. Linthicum, J. L. Small, K. M. Collins, C. J. Tucker, E. W. Pak, S. C. Britch, J. R. Eastman, J. E. Pinzon, K. L. Russell, Climate teleconnections and recent patterns of human and animal disease outbreaks. *PLoS Negl. Trop. Dis.* **6**, e1465 (2012).

27. S. Nabinejad, H. Schüttrumpf, Flood risk management in arid and semi-arid areas: A comprehensive review of challenges, needs, and opportunities. *Water* **15**, 3113 (2023).

28. D. W. Redding, R. Gibb, C. C. Dan-Nwafor, E. A. Ilori, R. U. Yashe, S. H. Oladele, M. O. Amedu, A. Iniobong, L. A. Attfield, C. A. Donnelly, I. Abubakar, K. E. Jones, C. Ihekweazu, Geographical drivers and climate-linked dynamics of Lassa fever in Nigeria. *Nat. Commun.* **12**, 5759 (2021).

29. R. Lowe, S. A. Lee, K. M. O'Reilly, O. J. Brady, L. Bastos, G. Carrasco-Escobar, R. De Castro Catão, F. J. Colón-González, C. Barcellos, M. S. Carvalho, M. Blangiardo, H. Rue, A. Gasparrini, Combined effects of hydrometeorological hazards and urbanisation on dengue risk in Brazil: A spatiotemporal modelling study. *Lancet Planet. Health* **5**, e209–e219 (2021).

30. D. Ekwem, J. Enright, J. G. C. Hopcraft, J. Buza, G. Shirima, M. Shand, J. K. Mwajombe, B. Bett, R. Reeve, T. Lembo, Local and wide-scale livestock movement networks inform disease control strategies in East Africa. *Sci. Rep.* **13**, 9666 (2023).

31. D. H. Braam, F. L. Jephcott, J. L. N. Wood, Identifying the research gap of zoonotic disease in displacement: A systematic review. *Glob. Health Res. Policy* **6**, 25 (2021).

32. J. C. Semenza, J. Rocklöv, K. L. Ebi, Climate change and cascading risks from infectious disease. *Infect. Dis. Ther.* **11**, 1371–1390 (2022).

33. R. K. Plowright, C. R. Parrish, H. McCallum, P. J. Hudson, A. I. Ko, A. L. Graham, J. O. Lloyd-Smith, Pathways to zoonotic spillover. *Nat. Rev. Microbiol.* **15**, 502–510 (2009).

34. R. McFarlane, A. Sleigh, A. McMichael, Land-use change and emerging infectious disease on an island continent. *Int. J. Environ. Res. Public Health* **10**, 2699–2719 (2013).

35. F. Keesing, L. K. Belden, P. Daszak, A. Dobson, C. D. Harvell, R. D. Holt, P. Hudson, A. Jolles, K. E. Jones, C. E. Mitchell, S. S. Myers, T. Bogich, R. S. Ostfeld, Impacts of

biodiversity on the emergence and transmission of infectious diseases. *Nature* **468**, 647–652 (2010).

36. M. P. Muehlenbein, "Human-wildlife contact and emerging infectious diseases" in *Human-Environment Interactions*, E. S. Brondízio, E. F. Moran, Eds. (Springer Netherlands, 2013), pp. 79–94; http://link.springer.com/10.1007/978-94-007-4780-7_4.

37. S. E. Randolph, A. D. M. Dobson, Pangloss revisited: A critique of the dilution effect and the biodiversity-buffers-disease paradigm. *Parasitology* **139**, 847–863 (2012).

38. J. P. Swaddle, S. E. Calos, Increased avian diversity is associated with lower incidence of human West Nile infection: Observation of the dilution effect. *PLOS ONE* **3**, e2488 (2008).

39. D. J. Salkeld, K. A. Padgett, J. H. Jones, A meta-analysis suggesting that the relationship between biodiversity and risk of zoonotic pathogen transmission is idiosyncratic. *Ecol. Lett.* **16**, 679–686 (2013).

40. WHO, "The Global Health Observatory: International Health Regulations (2005) States Parties Self-assessment Annual Report", 2024; https://www.who.int/data/gho/data/themes/international-health-regulations-(2005)-monitoring-framework.

41. M. Moore, B. Gelfeld, A. Okunogbe, C. Paul, *Identifying Future Disease Hot Spots: Infectious Disease Vulnerability Index* (RAND Corporation, 2016); www.rand.org/pubs/research_reports/RR1605.html.

42. L. O. Gostin, R. Katz, The international health regulations: The governing framework for global health security: The International Health Regulations. *Milbank Q.* **94**, 264–313 (2016).

43. GIDEON Informatics Inc., GIDEON (2023); www.gideononline.com/.

44. P. O. Title, J. B. Bemmels, ENVIREM: An expanded set of bioclimatic and topographic variables increases flexibility and improves performance of ecological niche modeling. *Ecography* **41**, 291–307 (2018).

45. I. Harris, T. J. Osborn, P. Jones, D. Lister, Version 4 of the CRU TS monthly high-resolution gridded multivariate climate dataset. *Sci. Data* **7**, 109 (2020).

46. M. Gilbert, G. Cinardi, D. Da Re, W. G. R. Wint, D. Wisser, T. P. Robinson, Gridded Livestock of the World - 2015 (GLW 4) (2022); https://dataverse.harvard.edu/dataverse/glw_4.

47. K. Winkler, R. Fuchs, M. Rounsevell, M. Herold, Global land use changes are four times greater than previously estimated. *Nat. Commun.* **12**, 2501 (2021).

48. P. Semenchuk, C. Plutzar, T. Kastner, S. Matej, G. Bidoglio, K.-H. Erb, F. Essl, H. Haberl, J. Wessely, F. Krausmann, S. Dullinger, Relative effects of land conversion and land-use intensity on terrestrial vertebrate diversity. *Nat. Commun.* **13**, 615 (2022).

49. A. J. Florczyk, C. Corbane, D. Ehrlich, S. Freire, T. Kemper, L. Maffenini, M. Melchiorri, M. Pesaresi, P. Politis, M. Schiavina, F. Sabo, L. Zanchetta, *GHSL Data Package 2019: Public Release GHS P2019* (Publications Office of the European Union, 2019).

50. D. J. Weiss, A. Nelson, C. A. Vargas-Ruiz, K. Gligorić, S. Bavadekar, E. Gabrilovich, A. Bertozzi-Villa, J. Rozier, H. S. Gibson, T. Shekel, C. Kamath, A. Lieber, K. Schulman, Y. Shao, V. Qarkaxhija, A. K. Nandi, S. H. Keddie, S. Rumisha, P. Amratia, R. Arambepola, E. G. Chestnutt, J. J. Millar, T. L. Symons, E. Cameron, K. E. Battle, S. Bhatt, P. W. Gething, Global maps of travel time to healthcare facilities. *Nat. Med.* **26**, 1835–1838 (2020).

51. H. A. Chipman, E. I. George, R. E. McCulloch, BART: Bayesian additive regression trees. *Ann. Appl. Stat.* **4**, 266–298 (2010).

52. M. Barbet-Massin, F. Jiguet, C. H. Albert, W. Thuiller, Selecting pseudo-absences for species distribution models: How, where and how many? *Methods Ecol. Evol.* **3**, 327–338 (2012).

53. W. J. Youden, Index for rating diagnostic tests. *Cancer* **3**, 32–35 (1950).

54. J. H. Friedman, Greedy function approximation: A gradient boosting machine. *Ann. Stat.* **29**, 1189–1232 (2001).

55. J. Kahlert, S. B. Gribsholt, H. Gammelager, O. M. Dekkers, G. Luta, Control of confounding in the analysis phase - An overview for clinicians. *Clin. Epidemiol.* **9**, 195–204 (2017).

56. M. E. Hopkins, C. L. Nunn, A global gap analysis of infectious agents in wild primates. *Divers. Distrib.* **13**, 561–572 (2007).

57. K. Yang, J. LeJeune, D. Alsdorf, B. Lu, C. K. Shum, S. Liang, Global distribution of outbreaks of water-associated infectious diseases. *PLoS Negl. Trop. Dis.* **6**, e1483 (2012).

58. S. J. Phillips, M. Dudík, J. Elith, C. H. Graham, A. Lehmann, J. Leathwick, S. Ferrier, Sample selection bias and presence-only distribution models: Implications for background and pseudo-absence data. *Ecol. Appl.* **19**, 181–197 (2009).

59. W. D. Fisher, On grouping for maximum homogeneity. *J. Am. Stat. Assoc.* **53**, 789–798 (1958).

60. R Core Team, A Language and Environment for Statistical Computing, R Foundation for Statistical Computing (2023); www.R-project.org.

61. V. Dorie, H. Chipman, R. McCulloch, dbarts: Discrete Bayesian Additive Regression Trees Sampler_. R package version 0.9-26 (2024); https://cran.r-project.org/web/packages/dbarts/index.html.

62. A. Fanelli, P. Tizzani, D. Buonavoglia, Crimean–Congo haemorrhagic fever (CCHF) in animals: Global characterization and evolution from 2006 to 2019. *Transbound. Emerg. Dis.* **69**, 1556–1567 (2022).

63. R. Vorou, Zika virus, vectors, reservoirs, amplifying hosts, and their potential to spread worldwide: What we know and what we should investigate urgently. *Int. J. Infect. Dis.* **48**, 85–90 (2016).

64. P.-G. U. Madueme, F. Chirove, Understanding the transmission pathways of Lassa fever: A mathematical modeling approach. *Infect. Dis. Model.* **8**, 27–57 (2023).

65. H. Li, J.-Y. V. Kim, B. S. Pickering, Henipavirus zoonosis: Outbreaks, animal hosts and potential new emergence. *Front. Microbiol.* **14**, 1167085 (2023).

66. M. Kwaśnik, W. Rożek, J. Rola, Rift Valley fever–A growing threat to humans and animals. *J. Vet. Res.* **65**, 7–14 (2021).

67. A. A. Rabaan, S. H. Al-Ahmed, S. Haque, R. Sah, R. Tiwari, Y. S. Malik, K. Dhama, M. I. Yatoo, D. K. Bonilla-Aldana, A. J. Rodriguez-Morales, SARS-CoV-2, SARS-CoV, and MERS-CoV: A comparative overview. *Infez. Med.* **1**, 174–184 (2020).

68. H.-J. Han, H. Yu, X.-J. Yu, Evidence for zoonotic origins of Middle East respiratory syndrome coronavirus. *J. Gen. Virol.* **97**, 274–280 (2016).

69. R. A. Mitu, M. R. Islam, The current pathogenicity and potential risk evaluation of marburg virus to cause mysterious "Disease X"—An update on recent evidences. *Environ. Health Insights* **18**, 11786302241235809 (2024).

70. K. Tsergouli, T. Karampatakis, A.-B. Haidich, S. Metallidis, A. Papa, Nosocomial infections caused by Crimean–Congo haemorrhagic fever virus. *J. Hosp. Infect.* **105**, 43–52 (2020).

71. A. T. Peterson, D. S. Carroll, J. N. Mills, K. M. Johnson, Potential mammalian filovirus reservoirs. *Emerg. Infect. Dis.* **10**, 2073–2081 (2004).

72. L. K. Koch, S. Cunze, J. Kochmann, S. Klimpel, Bats as putative *Zaire ebolavirus* reservoir hosts and their habitat suitability in Africa. *Sci. Rep.* **10**, 14268 (2020).

73. H. A. Hussein, Brief review on Ebola virus disease and one health approach. *Heliyon* **9**, e19036 (2023).

74. S. Rewar, D. Mirdha, Transmission of Ebola virus disease: An overview. *Ann. Glob. Health* **80**, 444 (2014).

75. L.-F. Wang, B. T. Eaton, "Bats, civets and the emergence of SARS," in *Wildlife and Emerging Zoonotic Diseases: The Biology, Circumstances and Consequences of Cross-*

Species Transmission, vol. 315 of *Current Topics in Microbiology and Immunology*, J. E. Childs, J. S. Mackenzie, J. A. Richt, Eds. (Springer Berlin Heidelberg, 2007), pp. 325–344.

76. M. Wang, M. Yan, H. Xu, W. Liang, B. Kan, B. Zheng, H. Chen, H. Zheng, Y. Xu, E. Zhang, H. Wang, J. Ye, G. Li, M. Li, Z. Cui, Y.-F. Liu, R.-T. Guo, X.-N. Liu, L.-H. Zhan, D.-H. Zhou, A. Zhao, R. Hai, D. Yu, Y. Guan, J. Xu, SARS-CoV infection in a restaurant from palm civet. *Emerg. Infect. Dis.* **11**, 1860–1865 (2005).

77. WHO, Marburg virus disease (2021); www.who.int/news-room/fact-sheets/detail/marburg-virus-disease.

78. R. G. Wallace, M. Gilbert, R. Wallace, C. Pittiglio, R. Mattioli, R. Kock, Did Ebola emerge in west Africa by a policy-driven phase change in agroecology? Ebola's social context. *Environ. Plan. Econ. Space* **46**, 2533–2542 (2014).
